# Riminophenazine Derivatives as Potential Antituberculosis Agents: Synthesis, Biological, and Electrochemical Evaluations

**DOI:** 10.3390/molecules26144200

**Published:** 2021-07-10

**Authors:** Mpelegeng Victoria Bvumbi, Chris van der Westhuyzen, Edwin M. Mmutlane, Andile Ngwane

**Affiliations:** 1 Department of Chemistry, University of Johannesburg, Auckland Park 2006, South Africa; edwinm@uj.ac.za; 2 CSIR, Pharmaceutical Technologies, Pretoria 0001, South Africa; cwvdwesthuyzen@csir.co.za; 3 Department of Chemistry, University of Venda, Private X5050, Thohoyandou 0950, South Africa; 4 Division of Molecular Biology and Human Genetics, Department of Biomedical Sciences, Faculty of Medicine and Health Sciences, Stellenbosch University, Tygerberg 7505, South Africa; andilengwane@gmail.com

**Keywords:** riminophenazines, alkyl substituent, drug discovery, *Mycobacterium tuberculosis*

## Abstract

A series of novel riminophenazine derivatives, having ionizable alkyl substituents at N-5 and a variety of substituents on the C-3 imino nitrogen, at C-8 and on the pendant aryl group, have been designed and synthesized. Preliminary investigations into the relationship between lipophilicity, redox potential, and antimycobacterial activity were conducted, using the in vitro activity against *Mycobacterium tuberculosis* H_37_Rv, mammalian cytotoxicity, and the redox potential of the compounds determined by cyclic voltammetry as measures. Results revealed an activity “cliff” associated with C-8 substitution (**10l** and **10m**) that, along with defined redox activity, point to a new class of riminophenazines as potential anti-tuberculosis agents having reasonable activity (MIC_99_ ~1 µM).

## 1. Introduction

**Figure 1:**
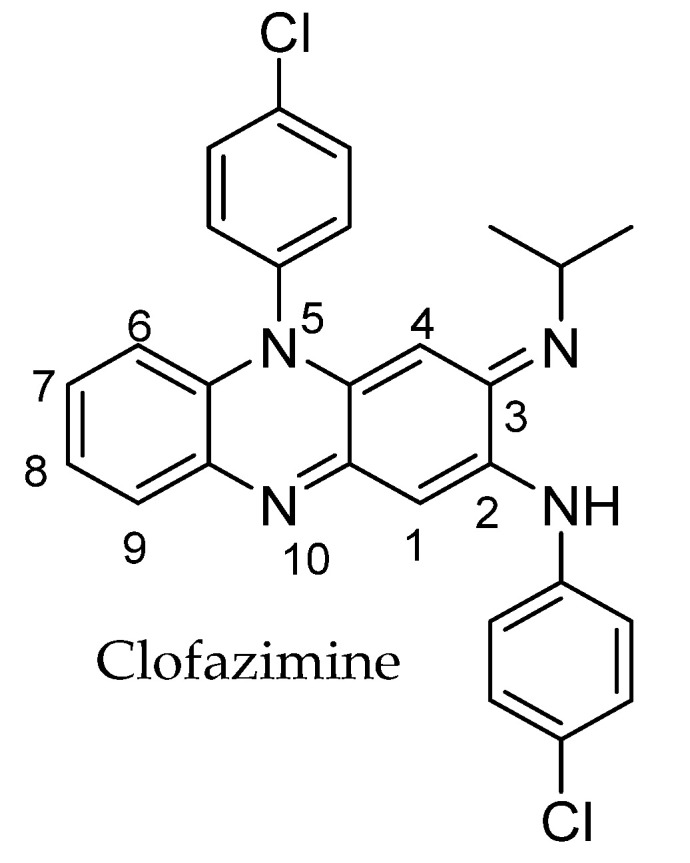
Structure of clofazimine.

Tuberculosis (TB), caused by *Mycobacterium tuberculosis* in man, continues to be a serious threat to public health [1]. Despite the extensive control measures in place, the disease burden still remains high globally, mainly as a result of multidrug-resistant strains and co-infection with HIV [2,3]. Additionally, approximately one-third of the world’s population have latent TB [4]. The disease occurs globally, with 22 countries bearing 80% of the total number of cases of active TB. This distribution occurs largely in the developing world, with sub-Saharan Africa being the worst affected [5,6]. An interesting class of tricyclic heterocycles, called riminophenazines, has shown promising activity against TB, leprosy, and cancer [7,8,9,10,11,12]. These compounds have a phenazine ring substituted on one of the ring nitrogens forming the central phenazine ring, as well as on one or both of the adjacent fused benzene rings. The designation “rimino” was coined to indicate the ‘R’ substituent on the imino moiety typical of this bioactive class of compounds [8]. This specific substituent is important in defining this bioactivity. A typical riminophenazine is clofazimine (Figure 1), which was first discovered by Barry et al. for the treatment of TB [13]. They proposed an intracellular redox cycling mechanism as the basis for the activity, in effect functioning as redox ‘traps’ that unproductively deplete cells of FADH and/or NAD(P)H. Clofazimine has also proved particularly active against *M. leprae*, which is the causative agent of leprosy [12,14,15]. Although the material (clofazimine) displayed high in vitro activity against TB, animal studies using guinea pig and monkey models failed to show in vivo activity [16]. Poor oral absorption of the drug has been implicated in certain studies, while species-specific activity may also be part of the problem based on considerable in vivo activity observed in hamsters and mice [17]. In vitro activity against the *M. avium complex* (MAC), an opportunistic pathogen in patients with HIV/AIDS, has also been demonstrated [18,19,20,21,22]. A major hindrance to the use of riminophenazines in medicine is the observed staining of the dermis and retina on prolonged use, which was likely as the result of deposition of these lipophilic materials in fatty tissues [11].

**Figure 2:**
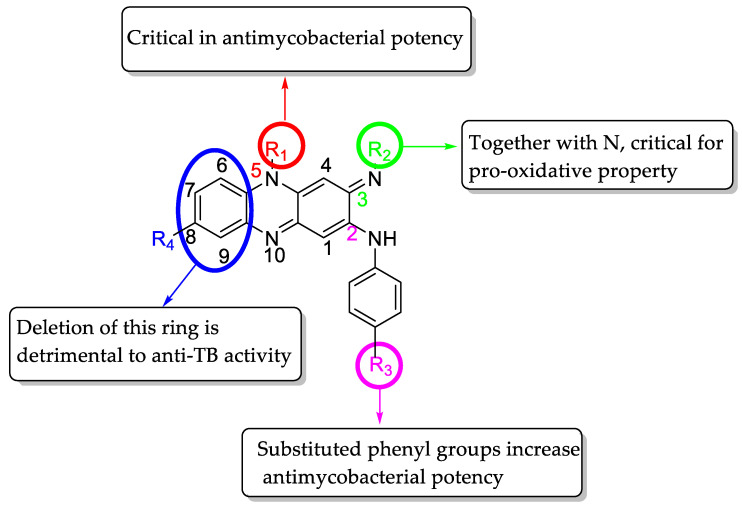
Structure–activity relationships of the riminophenazine system. IUPAC numbering is included for reference. Substituents targeted in this investigation are labeled R_1_–R_4_.

Previous SAR investigations using riminophenazines focused on varying the alkylimino group at C-3 and the phenyl group at N-5 as handles to improve activity and pharmacokinetic behavior [23,24,25,26]. Liu et al. proved that the phenazine nucleus is key to the activity of the materials [24]. In addition, while a phenyl substituent at N-5 is typical of bioactive riminophenazines, cycloalkyl groups are also well-tolerated [27]. The current state of SAR knowledge with respect to the riminophenazine compounds is summarized in Figure 2 [28].

With these facts and the foregoing SAR in mind, an investigation was undertaken to determine the impact of including an ionizable group at R_1_ on the riminophenazine skeleton on antimycobacterial activity. This may provide a means of solubilizing the material, in so doing diminishing accumulation in the fatty tissues of the skin. We particularly focused on using a limited number of short tertiary aminoalkyl chains for this purpose. In an attempt to optimize and/or modulate the impact of the amino moiety on function, a selection of substituents at R_2_–R_4_, having distinct physicochemical properties, were included for examination. The activity of these new compounds was assessed against the redox potential of the materials and their inherent lipophilicity.

## 2. Results and Discussion

### 2.1. Chemistry

**Scheme 1:**
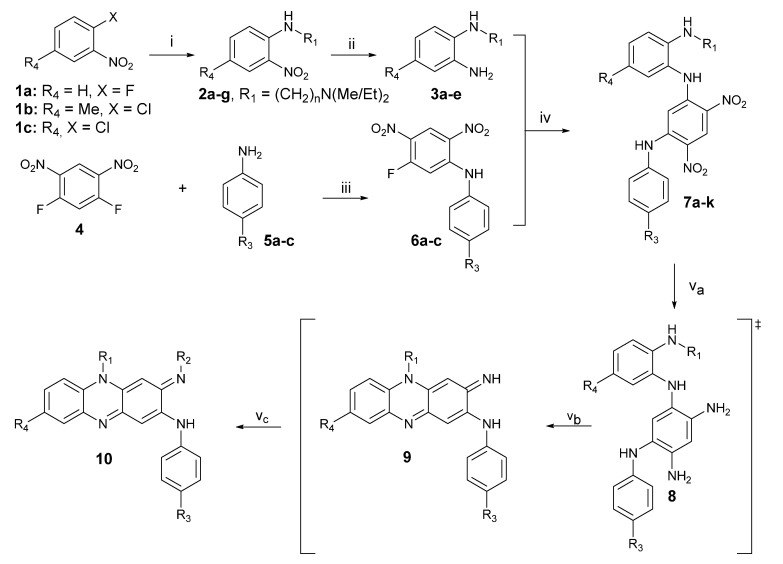
(i) R_1_ = Me_2_N(CH_2_)_2_: IPA or neat, 90–100 °C, 16–24 h, 74%; R_1_ = Me_2_N(CH_2_)_3_- or Et_2_N(CH_2_)_3_Et_2_NC_2_H_4_-: K_2_CO_3_, DMF, 90 °C, 24 h, 52–72%; (ii) 5% Pd/C, H_2_NNH_2_, 80 °C, 2–3 h; or 5% Pd/C, H_2_, rt, 4 h, 43–90%; (iii) 1,4-dioxane, Et_3_N, 70–80 °C, 4 h, 71–83%; (iv) DIPEA, dry EtOH, reflux, 16 h, 50–84%; (v) (a) Zn, AcOH, 50 °C, 1 h; or 5% Pd/C, H_2_, MeOH:THF, rt, 12 h; then (b) 5% Pd/C, H_2_, THF:MeOH, 12 h; then medical oxygen, MeOH, rt, 12 h; subdivide then (c) R_2_NH_2_, AcOH, dioxane, 110 °C, 48 h, 6–37%.

Several tertiary amines, in the form of *N*,*N*-diethyl- or *N*,*N*-dimethyl substituted ethylene- or propylenediamines, as well as *N*,*N*-dimethylhydrazine were chosen as candidates to generate the desired ionizable group at R_1_. Scheme 1 shows the synthetic sequence followed.

Three starting materials containing the ortho-halonitrobenzene structure **1** were commercially available, namely the parent compound, 1-fluoro-2-nitrobenzene, 1,4-dichloro-2-nitrobenzene, and 4-chloro-3-nitrotoluene. It was envisaged that the extra methyl or chloro group’s impact on lipophilicity might have a bearing on activity while also modulating the redox potential. *Ipso*-substitution of the halo groups ortho to the nitro moiety in these materials using each of the three aliphatic amines chosen proceeded smoothly to yield substituted 2-nitroanilines **2** [29]. Attempts to introduce *N*,*N*-dimethylhydrazine were not successful. Catalytic hydrogenation of the nitro function in **2** gave substituted 1,2-phenylenediamines **3** in reasonable yield [25], with the exception of the chloro analogues, for which facile protodehalogenation was noted under these conditions. This series was subsequently abandoned from the sequence. Concurrently, compounds **6** were synthesized by *ipso*-substitution of one of the fluorine groups of 1,5-difluoro-2,4-dinitrobenzene **4**. Three anilines **5** featuring a variety of para-substituents with varying electronic properties were used for this purpose, affording desired anilines **6**. Key intermediates **7** were obtained in modest yields by similar substitutions of each of the remaining fluoro groups in anilines **6** by the primary amino groups of phenylenediamines **3**. Reduction of the nitro groups in **7** under either acidic or neutral conditions, followed by immediate oxidative cyclization without prior isolation of tetramines **8**, afforded the desired iminophenazines **9** [25,30]. These latter two sets of compounds were not isolated because of difficulties in purification and questionable stability. Transimination of the unsubstituted imino moiety in compound **9** with commercially available isobutylamine, isopentylamine, or cyclohexylamine, was followed by column chromatography and preparative TLC to afford the target riminophenazines **10** in low yields [25,30].

### 2.2. Antimycobacterial Assessment

The compounds obtained were screened in triplicate against *M. tuberculosis* H_37_Rv using the MGIT 960 system at 1, 3, and 5 µM concentrations, with compounds showing MIC_99_ ≈1 µM further tested in triplicate in the range 0.0625–1 µM to refine the values determined. Cytotoxicity was determined using WI-38 cells (Human Fetal Lung Fibroblast) as a model of rapidly dividing normal mammalian cells. Although 26 compounds were synthesized, selected compounds were investigated, mainly due to the low quantities obtained synthetically. The data are summarized in Table 1.

Lipophilicity (LogP) is an important property for riminophenazine derivatives, as the skin discoloration side effect of clofazimine is closely related to its high lipophilicity. As shown in Table 1, the LogP values for the most new compounds are lower than that of clofazimine (7.50), ranging from 3.43 to 5.02. Modifications of the phenazine ring at position N-5 with alkyl diamines provided compounds having lower LogP values compared to clofazimine. Generally, replacement of the phenyl group at the N-5 position by an alkyl group lowered LogP values, depending on the structure of the alkyl groups. Compounds with a dimethylaminoethyl substituent at the N-5 position displayed significantly reduced lipophilicity as compared to the corresponding dimethylaminopropyl/diethylaminopropyl substituted compounds, as exemplified by compound **10o** (LogP 3.43) versus compound **10b** (LogP 4.21), compound **10g** (LogP 3.64) versus compound **10i** (LogP 3.74), and compound **10c** (LogP 4.31) versus compound **10f** (LogP 5.02). These results are consistent with previous observation made by Yi-li et al., who also observed that improved antibacterial activity is dependent on increasing lipophilicity, a major factor for skin pigmentation [27]. In addition, the benzyl substituent at the C-2 position with hydrogen, methyl, and ethoxy at the para position showed significantly reduced LogP values, with lipophilicity increasing as R_3_ was changes from H to OEt to Me. This was exemplified by compounds **10o**, **10n**, and **10g** with LogP values of (H: 3.43 < OEt: 3.64 < Me: 3.91). The same trend was observed for compounds **10a** (LogP 3.75), **10h** (LogP 3.96) and **10c** (LogP 4.31). **10o** could serve as an interesting lead for identifying new analogues with further decreased lipophilicity.

In terms of cytotoxicity, there are no general trends or variances with chain length or ring substitution. The R_2_ = cHex series in each case appears to be the least toxic set of compounds (compare **10d**, **10e** with **10c**, **10f**, or **10i**, **10j**, **10k** with **10g**, **10h**). Compounds in the R_3_ = OEt, R_4_ = H series tend to be among the least toxic members of each contiguous series. Further substitution of the system (compare **10g** and **10l**, **10f** and **10m**) increases the toxicity.

With regard to antimycobacterial action, most of the activity appears to be as a result of toxicity rather than selective bactericidal action. The exceptions are **10l, 10m**, and **10p** with both **10l** and **10m** having R_3_, R_4_ = Me as substituents, along with Me_2_N(CH_2_)_2_- at N-5. Similarly, **10p** has a methyl substituent at R_3_ which might also contribute to its activity. Compounds **10l, 10m**, and **10p** exhibited potent activity comparable to that of the control drug, isoniazid, and also the well-known riminophenazine drug, clofazimine, all with MIC values <1 µM. In each case, an activity cliff (MIC_99_ ≈ 1 μM) is encountered relative to all the other compounds, particularly conjoiners **10d** and **10e** (relative to **10l**) and **10c** and **10f** (relative to **10m**). The selectivity index of **10l, 10m**, and **10p** (defined as cytotoxicity IC_50_/MIC_99_), SI ≥ 3 is modest at best, but it does indicate that interesting activity is still possible using the ionizable groups included in the design. Most of the rest of the materials show an SI < 1.5 by comparison.

### 2.3. Cyclic Voltammetry Studies

**Figure 3:**
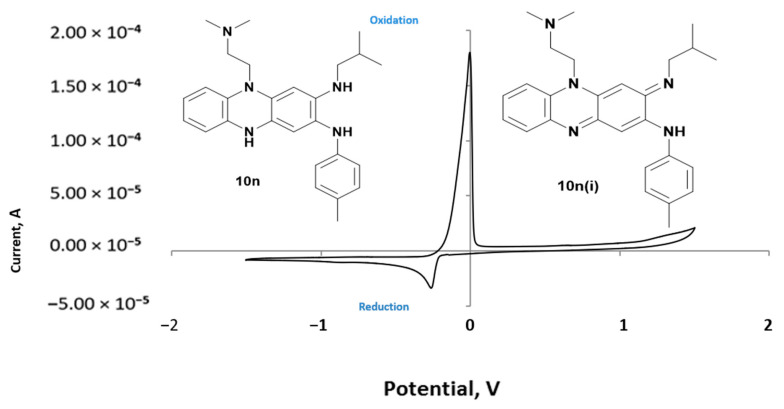
Cyclic voltammogram (CV) of **10n**.

Cyclic voltammetry was used to determine the redox potential of the compounds, as a measure of the electronic impact the various substituents introduced on the riminophenazine skeleton may have. The results are summarized in Table 2. The cyclic voltammograms of the compounds each displayed a fully reversible one-electron transfer process. For example, the cyclic voltammogram of **10n** (Figure 3) showed that the redox cycle was fully reversible: increasingly negative (reducing) applied potential through a minimum anodic current produced reduced species on the left (**10n**), while an increasingly positive (oxidative) potential produces a maximum cathodic current to afford the fully oxidized species [**10n(i)**] to the right. All the compounds had negative half-wave potentials, indicating that these riminophenazines are easily oxidized—as observed in the laboratory [31].

It is interesting to note that the half-wave potential of the compounds proved to be a modest indicator of activity. All the compounds having MIC99 < 3 μM showed *E*_½_ values between −0.20 V and −0.13 V. Compounds (**10b**, **10c**, **10e**, **10f**, **10j**) with *E*_½_ > −0.22 V were generally inactive. This may indicate, in conjunction with the structural requirements for the observed activity cliff, that there is both an electronic/redox potential aspect, as well as some form of specific steric/binding determinant operative in this series of compounds that may be further exploited to improve the observed activity.

## 3. Materials and Methods 

The reactions were monitored for their completion by thin layer chromatography (TLC) using aluminium-backed Macherey-Nagel ALUGRAM Sil G/UV254 plates pre-coated with 0.25 mm silica gel 60Å. Column chromatography was carried out on silica gel 60 (particle size 200–300 mesh) with a silica to compound ratio of 30:1 by mass or neutral alumina as the adsorbent for conventional preparative chromatography.

NMR spectra were recorded on a Varian INOVA 400 MHz system or a Varian PremiumShield VNMRS 600 MHz system operating at 399.94 or 599.74 MHz, respectively. Chemical shifts (δ) are reported in parts per million (ppm) relative to tetramethylsilane (TMS) or against the residual protonated solvent signal as reference. Coupling constants (J) are reported in Hz. 

Melting points were obtained on a Reichert Hot Stage or a Mettler FP62 melting point apparatus. High-resolution mass spectra were recorded on a Waters Acquity UPLC coupled in tandem to a Waters photodiode array (PDA) detector and a SYNAPT G1 HDMS mass spectrometer.

### 3.1. Synthesis Procedures

#### General Procedure for Final Compounds **10a–p**

The chosen dinitro compound **7** (3 mmol) and 5% palladium on carbon (3 mmol) in 2:3 (*v*/*v*) methanol:THF was stirred at room temperature under 275 kPa hydrogen atmosphere for 12 h in a steel pressure vessel. The orange solution turned maroon after the completion. The catalyst was filtered off through a celite pad, and the filtrate was concentrated under reduced pressure. The gummy maroon residue was dissolved in methanol and stirred at room temperature under pure oxygen (99.5%) atmosphere (345 kPa) for 12 h. The mixture was concentrated under reduced pressure to give a dark red/black product. The crude residue was purified on silica using 5–15% methanol:chloroform as eluent and used immediately without characterization. The material obtained was divided among a choice of amines (isobutylamine, cyclohexylamine, or isopentylamine) (25 eq.) in dioxane (3–4 mL) and glacial acetic acid (0.2 eq.) and heated at 110 °C in a sealed or closed tube for 48 h. After cooling to room temperature, the material was concentration in vacuo, and the residue obtained was purified by column chromatography on silica gel [2–5% (*v*/*v*) methanol:chloroform as eluent]. Further purification using preparative TLC was required using the same eluent to ensure adequate purity of the isolated materials.

*(E)-5-[2-(Dimethylamino)ethyl]-3-cyclohexylimino-2-phenylamino-3,5-dihydrophenazine* (**10a**). Dark red solid (20.4 mg, 20.4%); *R*_f_ 0.46 [10% (*v*/*v*) methanol: chloroform]; mp. 94–96 °C (ethanol). ^1^H NMR (400 MHz, CD_3_OD): δ 7.33 (dd, *J* = 7.93, 1.03 Hz, 1H, H 9), 7.27 (dd, *J* = 8.51, 7.78 Hz, 2H, H 3’), 7.21–7.17 (m, H 2’), 7.15 (d, *J* = 7.70 Hz, 1H, H 6), 7.11 (d, *J* = 8.48 Hz, 1H, H 7), 6.99 (dt, *J* = 13.1, 7.3 Hz, 2H, H 8), 6.53 (s, 1H, H 1), 5.74 (s, 1H, H 4), 3.91 (br s, 2H, NCH_2_CH_2_), 3.48–3.36 (m, 1H, =NCH), 2.46–2.35 (m, 2H, CH_2_NMe_2_), 2.26 (s, 6H, NMe_2_), 1.93–1.75, 1.75–1.63, 1.49–1.34, and 1.33–1.24 [4 × m, 10H, NCH(C_5_H_10_)]; ^13^C NMR (101 MHz, CD_3_OD): δ 152.4 (C 3), 151.7 (C-10a), 145.1 (C 2), 141.4 (C 1’), 136.7 (C 9a), 134.7 (C-4a), 131.5 (C 5a), 130.6 (C 3’), 129.7 (C 4’), 128.9 (C 9), 124.7 (C 7), 124.2 (C 8), 122.4 (C 2’), 114.0 (C 6), 99.9 (C 1), 89.0 (C 4), 59.3 (=NCH), 54.8 (CH_2_NMe_2_), 46.1 (NMe_2_), 44.8 (NCH_2_), 35.0 [CH(CH_2_)_2_], 27.2 [CH_2_(CH_2_)_2_], 26.3 [CH_2_(CH_2_)_2_]; HRMS (ESI-TOF+): *m*/*z* calculated for C_28_H_34_N_5_: 440.2814; found: 440.2810 (MH+). IR (cm^−1^): 3209 (N-H), 2923 (Ar-C-H), 1508 (C=N), 1463 (Ar-C=C), 1241 (C-N).

*(E)-5-[3-(Diethylamino)propyl]-3-isobutylimino-2-phenylamino-3,5-dihydrophenazine* (**10b**). Dark red solid (12.5 mg, 37%); *R*_f_ 0.42 [20% (*v*/*v*) methanol: chloroform]; mp. 102–104 °C (ethanol). ^1^H NMR (400 MHz, CD_3_OD): δ 8.19 (br d, *J* = 8.79 Hz, 1H, H 6), 8.03 (dd, *J* = 8.35, 1.42 Hz, 1H, H 9), 7.89 (ddd, *J* = 8.76, 7.10, 1.51 Hz, 1H, H 7), 7.70 (ddd, *J* = 8.18, 7.10, 0.93 Hz, 1H, H 8), 7.50–7.45 (m, 2H, H 3′), 7.41–7.37 (m, 2H, H 2′), 7.29 (s, 1H, H 1), 7.23 (ddt, *J* = 7.30, 7.23, 1.17 Hz, 1H, H 4′), 6.77 (s, 1H, H 4), 4.93 (br t, *J* = 7.62 Hz, 2H, NCH_2_CH_2_), 3.54 (d, *J* = 7.13 Hz, 2H, NCH_2_CH), 2.88 (t, *J* = 7.08 Hz, 2H, CH_2_NEt_2_), 2.73 [q, *J* = 7.16 Hz, 4H, N(CH_2_CH_3_)_2_], 2.29 (dquin, *J* = 13.49, 6.78 Hz, 1H, CHMe_2_), 2.17 (dt, *J* = 15.11, 7.43 Hz, 2H, -CH_2_-), 1.16 (d, *J* = 6.64 Hz, 6H, CHMe_2_) and 1.15 [t, *J* = 7.13 Hz, 6H, N(CH_2_CH_3_)_2_]; ^13^C NMR (101 MHz, CD_3_OD): 154.4 (C 3), 147.2 (C 10a), 142.6 (C 2), 141.3 (C 1′), 140.4 (C 9a), 135.5 (C 4a), 133.7 (C 7), 131.5 (C 9), 131.0 (C 3′), 130.7 (C 5a), 128.5 (C 8), 126.2 (C 4′), 123.7 (C 2′), 117.2 (C 6), 107.7 (C 1), 90.2 (C 4), 53.9 (NCH_2_CH), 50.6 (CH_2_NEt_2_), 48.3 [N(CH_2_CH_3_)_2_], 47.6 (NCH_2_CH_2_), 29.2 (CHMe_2_), 25.7 (-CH_2_-), 21.2 (CHMe_2_) and 11.6 [N(CH_2_CH_3_)_2_]. HRMS (ESI-TOF+): *m*/*z* calculated for C_29_H_38_N_5_: 456.3127; found: 456.3114 (MH+). IR (cm^−1^): 3381 (N-H), 2980 (Ar-C-H), 1509 (C=N), 1461 (Ar-C=C), 1250 (C-N).

*(E)-5-[2-(Dimethylamino)ethyl]-3-cyclohexylimino-2-(4-methylphenyl)amino-3,5-dihydrophenazine* (**10c**). Maroon solid (38 mg, 12% yield); *R*_f_ 0.39 [10% (*v*/*v*) methanol: chloroform]; mp. 110–112 °C (ethanol). ^1^H NMR (400 MHz, CD_3_OD): δ 7.39 (dd, *J* = 7.91, 1.37 Hz, 1H, H 6), 7.19 (br d, *J* = 7.70 Hz, 1H, H 9), 7.24 (ddd, *J* = 8.40, 7.03, 1.37 Hz, 1H, H 7), 7.12 (d, *J* = 8.49 Hz, 2H, H 2’), 7.10 (d, *J* = 8.89 Hz, 2H, H 3’), 7.04 (ddd, *J* = 8.01, 6.93, 1.07 Hz, 1H, H 8), 6.53 (s, 1H, H 1), 5.82 (s, 1H, H 4), 4.00 (br t, *J* = 7.30 Hz, 2H, NCH_2_CH_2_), 3.50 (br td, 1H, =NCH), 2.50 (br t, *J* = 7.81 Hz, 2H, CH_2_NMe_2_), 2.34 (s, 6H, NMe_2_), 2.29 (3H, s, C-4’ Me), 1.93–1.75, 1.75–1.63, and 1.53–1.35 [10H, 3 × m, NCH(C5H10)]; ^13^C NMR (101 MHz, CD_3_OD): δ 152.4 (C 3), 151.6 (C-10a), 145.4 (C 2), 138.6 (C 9a), 136.8 (C 1’), 134.6 (C 4’), 134.0 (C 4a), 131.3 (C 7), 131.1 (C 3’), 129.6 (C 9), 128.8 (C 5a), 124.2 (C 8), 122.6 (C 2’), 113.9 (C 6), 99.5 (C 1), 89.0 (C 4), 59.2 (=NCH), 54.8 (CH_2_NMe_2_), 46.1 (NMe_2_), 44.8 (NCH_2_), 35.0 [CH(CH_2_)_2_], 27.2 [CH_2_(CH_2_)_2_], 26.3 (C-4’ Me), 21.1 [CH_2_(CH_2_)_2_]; HRMS (ESI-TOF+): *m*/*z* calculated for C_29_H_36_N_5_: 454.2971; found: 454.2956 (MH+); IR (cm^−1^): 3292 (N-H), 2920 (Ar-C-H), 1517 (C=N) and 1465 (Ar-C=C), 1240 (C-N).

*(E)-5-[3-(Dimethylamino)propyl]-3-isobutylimino-2-(4-methylphenyl)amino-3,5-dihydrophenazine* (**10d**). Dark maroon crystals (49 mg; 13%); *R*_f_ 0.30 [20% (*v*/*v*) methanol:chloroform]; mp. > 230 °C (ethanol). ^1^H NMR (600 MHz, CD_3_OD): δ 8.25 (br d, *J* = 8.30 Hz, 1H, H 6), 8.23 (d, *J* = 7.90, Hz, 1H, H 9), 8.08 (t, *J* = 7.52 Hz, 1H, H 7), 7.90 (t, *J* = 7.50 Hz, 1H, H 8), 7.66 (d, *J* = 8.25 Hz, 2H, H 3′), 7.40 (d, *J* = 8.40 Hz, 2H, H 2′), 7.40 (s, 1H, H 1), 6.88 (s, 1H, H 4), 5.03 (br t, *J* = 7.63 Hz, 2H, NCH_2_CH_2_), 3.80 (d, *J* = 7.04 Hz, 2H, NCH_2_CH), 2.93 (t, *J* = 6.90 Hz, 2H, CH_2_NMe_2_), 2.77 (s, 6H, NMe_2_), 2.71 (s, C-4′ 3H, Me), 2.58 (quin, J = 7.04 Hz, CHMe_2_), 2.44 (dt, *J* = 15.26, 7.34 Hz, 2H, -CH_2_-) and 1.52 (d, *J* = 6.75 Hz, 6H, CHMe_2_); ^13^C NMR (151 MHz, CD_3_OD): 154.4 (C 3), 147.0 (C 10a), 142.8 (C 2), 140.7 (C 9a), 138.4 (C 1′), 136.6 (C 4′), 135.6 (C 4a), 133.8 (C 7), 131.7 (C 9), 131.6 (C 3′), 130.6 (C 5a), 128.8 (C 8), 124.3 (C 2′), 117.4 (C 6), 107.4 (C 1), 90.3 (C 4), 57.1 (CH_2_NMe_2_), 53.4 (NCH_2_CH), 47.5 (NMe_2_), 45.6 (NCH_2_CH_2_), 29.1 (CHMe_2_), 26.1 (-CH_2_-), 21.2 (C-4′ Me) and 21.1 (CHMe_2_); HRMS (ESI-TOF+): *m*/*z* calculated for C_28_H_36_N_5_: 442.2971; found: 442.2892 (MH+). IR (cm^−1^): 3108 (N-H), 2954 (Ar-C-H), 1512 (C=N), 1459 (Ar-C=C), 1239 (C-N).

*(E)-5-[3-(Diethylamino)propyl]-3-isobutylimino-2-(4-methylphenyl)amino-3,5-dihydrophenazine* (**10e**). Dark red solid (22.4 mg, 5.8%); *R*_f_ 0.38 [10% (*v*/*v*) methanol:chloroform]; mp. 80–82 °C (ethanol). ^1^H NMR (400 MHz, CD_3_OD): δ 8.11 (br d, *J* = 8.89 Hz, 1H, H 6), 7.99 (br d, *J* = 8.30 Hz, 1H, H 9), 7.82 (br t, 1H, *J* = 7.80 Hz, H 7), 7.64 (br t, *J* = 7.80 Hz, 1H, H 8), 7.27 (q, *J* = 8.89 Hz, 4H, H 3′, H 2′), 7.17 (s, 1H, H 1), 6.68 (s, 1H, H 4), 4.84 (br t, *J* = 8.30 Hz, 2H, NCH_2_CH_2_), 3.49 (d, *J* = 7.03 Hz, 2H, NCH_2_CH), 2.76 (t, *J* = 6.93 Hz, 2H, CH_2_NEt_2_), 2.64 [q, *J* = 7.13 Hz, 4H, N(CH_2_CH_3_)_2_], 2.38 (s, 3H, C-4′ Me), 2.25 (dquin, *J* = 13.54, 6.75 Hz, 1H, CHMe_2_), 2.15–2.06 (m, *J* = 8.50, 2H, -CH_2_-), 1.13 [d, *J* = 7.18 Hz, 6H, N(CH_2_CH_3_)_2_], and 1.10 (t, *J* = 6.64 Hz, 6H, CHMe_2_); ^13^C NMR (101 MHz, CD_3_OD): 154.2 (C 3), 147.9 (C 10a), 143.9 (C 2), 140.1 (C 9a), 138.5 (C 1′), 136.3 (C 4′), 135.3 (C 4a), 133.1 (C 7), 131.2 (C 9), 131.5 (C 3′), 130.7 (C 5a), 128.0 (C 8), 124.0 (C 2′), 116.9 (C 6), 107.0 (C 1), 90.4 (C 4), 54.0 (NCH_2_CH), 51.0 (CH_2_NEt_2_), 48.3 [N(CH_2_CH_3_)_2_], 47.5 (NCH_2_CH_2_), 29.5 (CHMe_2_), 26.1 (-CH_2_-), 21.4 (CHMe_2_), 21.3 (C-4′ Me) and 11.9 [N(CH_2_CH_3_)_2_]; HRMS (ESI-TOF+): *m*/*z* calculated for C_30_H_40_N_5_: 470.3284; found: 470.3291 (MH+); IR (cm^−1^): 3367 (N-H), 2980 (Ar-C-H), 1517 (C=N), 1464 (Ar-C=C), 1250 (C-N).

*(E)-5-[3-(Diethylamino)propyl]-3-cyclohexylimino-2-(4-methylphenyl)amino-3,5-dihydrophenazine* (**10f**). Dark red solid (35.9 mg, 9.7%); *R*_f_ 0.36 [20% (*v*/*v*) methanol:chloroform]; mp. 108–110 °C (ethanol). ^1^H NMR (400 MHz, CD_3_OD): δ 8.19 (br d, *J* = 8.79 Hz, 1H, H 6), 8.04 (dd, *J* = 8.40, 1.37 Hz, 1H, H 9), 7.87 (ddd, *J* = 8.69, 7.13, 1.57 Hz, 1H, H 7), 7.70 (ddd, *J* = 8.30, 7.13, 0.88 Hz, 1H, H 8), 7.29 (d, *J* = 8.01 Hz, 2H, H 3’), 7.26 (d, *J* = 9.18 Hz, 2H, H 2’), 7.22 (s, 1H, H 1), 6.81 (s, 1H, H 4), 4.98–4.88 (m, 2H, NCH_2_), 4.07 (br t, *J* = 7.81 Hz, 1H, =NCH), 2.81 (t, *J* = 7.13 Hz, 2H, CH_2_NEt_2_), 2.67 [q, *J* = 7.10 Hz, 4H, N(CH_2_CH_3_)_2_], 2.38 (s, 3H, C-4’ Me), 2.23–2.17 (m, 2H, -CH_2_-), 1.82–1.18 [4 × m, 10H, NCH(C_5_H_10_)], and 1.10 [t, *J* = 7.23 Hz, 6H, N(CH_2_CH_3_)_2_]; ^13^C NMR (101 MHz, CD_3_OD): δ 152.8 (C 3), 147.3 (C-10a), 143.2 (C 2), 140.5 (C 9a), 138.4 (C 1’), 136.6 (C 4’), 135.5 (C 4a), 133.4 (C 7), 131.6 (C 3’), 131.5 (C 9), 130.6 (C 5a), 128.5 (C 8), 124.3 (C 2’), 117.2 (C 6), 106.8 (C 1), 90.1 (C 4), 55.3 (=NCH), 50.9 (CH_2_NEt_2_), 48.3 [N(CH_2_CH_3_)_2_], 47.6 (NCH_2_), 33.1 [CH(CH_2_)_2_], 26.7 (-CH_2_-), 26.2 [CH_2_(CH_2_)_2_], 21.1 (C-4’ Me) and 11.8 [N(CH_2_CH_3_)_2_]; HRMS (ESI-TOF+): *m*/*z* calculated for C_32_H_42_N_5_: 496.3440; found: 496.3460 (MH+). IR (cm^−1^): 3367 (N-H), 2980 (Ar-C-H), 1511 (C=N), 1462 (Ar-C=C), 1250 (C-N). 

*(E)-5-[2-(Dimethylamino)ethyl]-3-isobutylimino-2-(4-ethoxyphenyl)amino-3,5-dihydrophenazine* (**10g**). Dark red solid (193 mg, 47%); *R*_f_ 0.32 [10% (*v*/*v*) methanol:chloroform]; mp. 208–210 °C (ethanol). ^1^H NMR (400 MHz, CD_3_OD): δ 8.09 (br d, *J* = 8.98 Hz, 1H, H 6), 8.04 (br d, *J* = 7.81 Hz, 1H, H 9), 7.83 (dd, *J* = 8.49, 6.93 Hz, 1H, H 7), 7.65 (t, *J* = 7.42 Hz, 1H, H 8), 7.29 (d, *J* = 8.79 Hz, H 2’), 7.09 (d, *J* = 8.89 Hz, 2H, H 3’), 7.03 (s, 1H, H 1), 6.87 (s, 1H, H 4), 4.90 (dd, *J* = 7.42, 6.54 Hz, 2H, NCH_2_CH_2_), 4.09 (q, *J* = 6.97 Hz, 2H, OCH_2_CH_3_), 3.52 (d, *J* = 7.0 Hz, 2H, NCH_2_CH), 2.89 (t, *J* = 7.37 Hz, 2H, CH_2_NMe_2_), 2.46 (s, NMe_2_), 2.26 (sep, *J* = 6.63 Hz, 1H, CHMe_2_), 1.43 (t, *J* = 6.98 Hz, 3H, OCH_2_CH_3_), 1.14 (d, *J* = 6.64 Hz, 6H, CHMe_2_); ^13^C NMR (101 MHz, CD3OD): δ 158.5 (C 4’), 154.1 (C 3), 147.2 (C 10a), 143.5 (C 2), 140.4 (C 9a), 135.5 (C 4a), 133.3 (C 1’), 133.1 (C 7), 131.3 (C 9), 130.4 (C 5a), 128.5 (C 8), 126.4 (C 2’), 116.9 (C 3’), 116.8 (C 6), 105.7 (C 1), 90.3 (C 4), 64.9 (OCH_2_CH_3_), 56.6 (CH_2_NMe_2_), 53.5 (NCH_2_CH), 47.9 (NCH_2_CH_2_), 46.1 (NMe_2_), 28.9 (CHMe_2_), 20.9 (CHMe_2_) and 15.2 (OCH_2_CH_3_); HRMS (ESI-TOF+): *m*/*z* calculated for C_28_H_36_N_5_O: 458.2920; found: 458.2891 (MH+); IR (cm^−1^): 3128 (N-H), 2954 (Ar-C-H), 1507 (C=N), 1464 (Ar-C=C), 1230 (C-N). 

*(E)-5-[2-(Dimethylamino)ethyl]-3-cyclohexylimino-2-(4-ethoxyphenyl)amino-3,5-dihydrophenazine* (**10h**). Dark red solid (45 mg, 14%); *R*_f_ 0.42 [10% (*v*/*v*) methanol:chloroform]; mp. 222–224 °C (ethanol). ^1^H NMR (400 MHz, CD_3_OD): δ 8.04–7.96 (m, 2H, H 6, H-9), 7.82 (dd, *J* = 11.5, 4.2 Hz, 1H, H 7), 7.65 (br t, *J* = 7.45 Hz, 1H, H 8), 7.30–7.25 (m, 2H, H 2’), 7.04 (s, 1H, H 1), 7.02 (d, *J* = 8.80 Hz, 2H, H 3’), 6.83 (s, 1H, H 4), 4.85 (obscured, 2H, NCH_2_), 4.08 (q, *J* = 6.85 Hz, 2H, OCH_2_CH_3_), 3.92 (m, 2H, =NCH), 2.85 (t, *J* = 7.63 Hz, 2H, CH_2_NMe_2_), 2.46 (s, 6H, NMe_2_), 2.23–2.15, 1.96–1.85, 1.83–1.75 and 1.62–1.55 [4 × m, 10H, NCH(C_5_H_10_)], 1.40 (t, *J* = 6.90 Hz, 3H, OCH_2_CH_3_); ^13^C NMR (101 MHz, CD_3_OD): δ 158.6 (C 4’), 152.7 (C 3), 147.7 (C 10a), 144.1 (C 2), 140.2 (C 9a), 135.6 (C 4a), 133.3 (C 7), 133.2 (C 1’), 131.1 (C 9), 130.6 (C 5a), 128.2 (C 8), 126.6 (C 2’), 117.0 (C 3’), 116.7 (C 6), 105.1 (C 1), 90.4 (C 4), 65.1 (OCH_2_CH_3_), 56.8 (=NCH), 55.8 (CH_2_NMe_2_), 47.7 (NCH_2_), 46.3 (NMe_2_), 33.3 [CH(CH_2_)_2_], 26.7 and 26.2 [CH_2_(CH_2_)_2_] and 15.3 (OCH_2_CH_3_); HRMS (ESI-TOF+): *m*/*z* calculated for C_30_H_38_N_5_O: 484.3076; found: 484.3048 (MH+).

*(E)-5-[3-(Dimethylamino)propyl]-3-isobutylimino-2-(4-ethoxyphenyl)amino-3,5-dihydrophenazine* (**10i**). Dark red solid (41.0 mg, 15%); *R*_f_ 0.40 [20% (*v*/*v*) methanol:chloroform]; mp. 185–187 °C (ethanol). ^1^H NMR (400 MHz, CD_3_OD): δ 8.21 (d, *J* = 8.79 Hz, 1H, H 6), 8.05 (d, *J* = 8.40 Hz, 1H, H 9), 7.84 (ddd, *J* = 9.00, 7.19, 1.27 Hz, 1H, H 7), 7.72 (t, *J* = 7.70 Hz, 1H, H 8), 7.30 (d, *J* = 8.89 Hz, 2H, H 2’), 7.11 (s, 1H, H 1), 7.03 (d, *J* = 8.98 Hz, 2H, H 3’), 6.87 (s, 1H, H 4), 4.98 (t, *J* = 7.80 Hz, 2H, NCH_2_CH_2_), 4.08 (q, *J* = 6.93 Hz, 2H, OCH_2_CH_3_), 3.55 (d, *J* = 7.03 Hz, 2H, NCH_2_CH), 2.61 (t, *J* = 6.83 Hz, 2H, CH_2_NMe_2_), 2.34 (s, 6H, NMe_2_), 2.27 (sep, *J* = 6.77 Hz, 1H, CHMe_2_), 2.18 (br t, *J* = 6.74 Hz, 2H, -CH_2_-), 1.41 (t, *J* = 6.98 Hz, 3H, OCH_2_CH_3_), 1.15 (d, *J* = 6.64 Hz, 6H, CHMe_2_); ^13^C NMR (101 MHz, CD_3_OD): δ 158.7 (C 4’), 154.1 (C 3), 147.2 (C 10a), 143.7 (C 2), 140.6 (C 9a), 135.5 (C 4a), 133.5 (C 1’), 133.3 (C 7), 131.5 (C 9), 130.5 (C 5a), 128.7 (C 8), 126.6 (C 3’), 117.3 (C 6), 117.0 (C 2’), 106.0 (C 1), 90.3 (C 4), 65.1 (OCH_2_CH_3_), 57.3 (CH_2_NMe_2_), 53.6 (NCH_2_CH), 47.6 (NCH_2_CH_2_), 46.0 (NMe_2_), 29.1 (CHMe_2_), 26.4 (-CH_2_-), 21.1 (CHMe_2_), and 15.3 (OCH_2_CH_3_); HRMS (ESI-TOF+): *m*/*z* calculated for C_29_H_38_N_5_O: 472.3076; found: 472.3048 (MH+); IR (cm^−1^): 3085 (N-H), 2955 (Ar-C-H), 1502 (C=N), 1462 (Ar-C=C), 1232 (C-N).

*(E)-5-[3-(Diethylamino)propyl]-3-isobutylimino-2-(4-ethoxyphenyl)amino-3,5-dihydrophenazine* (**10j**). Dark purple solid (22.1 mg, 6.2%); *R*_f_ 0.37 [20% (*v*/*v*) methanol:chloroform]; mp. 205–207 °C (ethanol). ^1^H NMR (400 MHz, CD_3_OD): δ 7.98 (d, *J* = 8.79 Hz, 1H, H 6), 7.84 (dd, *J* = 8.24, 1.28 Hz, 1H, H 9), 7.69 (ddd, *J* = 8.63, 7.17, 1.40 Hz, 1H, H 7), 7.52 (ddd, *J* = 8.20, 6.95, 0.70 Hz, 1H, H 8), 7.16 (d, *J* = 9.03 Hz, 2H, H 2’), 6.91 (s, 1H, H 1), 6.88 (d, *J* = 8.91 Hz, 2H, H 3’), 6.55 (s, 1H, H 4), 4.71 (t, *J* = 7.80 Hz, 2H, NCH_2_CH_2_), 3.99 (q, *J* = 7.00 Hz, 2H, OCH_2_CH_3_), 3.39 (d, *J* = 7.08 Hz, 2H, NCH_2_CH), 2.66 (t, *J* = 6.96 Hz, 2H, CH_2_NEt_2_), 2.55 [q, *J* = 7.12 Hz, 4H, N(CH_2_CH_3_)_2_], 2.14 (sep, *J* = 6.76 Hz, 1H, CHMe_2_), 2.03–1.94 (m, 2H, -CH_2_-), 1.29 (t, *J* = 7.02 Hz, 3H, OCH_2_CH_3_), 1.01 (d, *J* = 6.59 Hz, 6H, CHMe_2_), and 0.97 [t, *J* = 7.14 Hz, 6H, N(CH_2_CH_3_)_2_]; ^13^C NMR (101 MHz, CD_3_OD): δ 158.5 (C 4’), 154.0 (C 3), 148.0 (C 10a), 144.2 (C 2), 139.9 (C 9a), 135.5 (C 4a), 133.4 (C 1’), 132.7 (C 7), 130.9 (C 9), 130.6 (C 5a), 127.8 (C 8), 126.3 (C 3’), 116.9 (C 2’), 116.8 (C 6), 104.6 (C 1), 89.9 (C 4), 65.1 (OCH_2_CH_3_), 54.7 (CH_2_NEt_2_), 50.9 (NCH_2_CH), 48.3 [N(CH_2_CH_3_)_2_], 47.4 (NCH_2_CH_2_), 29.5 (CHMe_2_), 25.8 (-CH_2_-), 21.2 (CHMe_2_), 15.3 (OCH_2_CH_3_) and 11.9 [N(CH_2_CH_3_)_2_]; HRMS (ESI-TOF+): *m*/*z* calculated for C_31_H_42_N_5_O: 500.3389; found: 500.3391 (MH+); IR (cm^−1^): 3365 (N-H), 2980 (Ar-C-H), 1509 (C=N), 1462 (Ar-C=C), 1233 (C-N).

*(E)-5-[3-(Diethylamino)propyl]-3-isopentylimino-2-(4-ethoxyphenyl)amino-3,5-dihydrophenazine* (**10k**). Dark purple solid (19.1 mg, 6.2%) after crystallization; *R*_f_ 0.39 [20% (*v*/*v*) methanol:chloroform]; mp. 198–200 °C (ethanol). ^1^H NMR (400 MHz, CD_3_OD): δ 8.17 (d, *J* = 8.79 Hz, 1H, H 6), 8.02 (dd, *J* = 8.30, 1.37 Hz, 1H, H 9), 7.85 (ddd, *J* = 8.74, 7.13, 1.42 Hz, 1H, H 7), 7.69 (ddd, *J* = 8.30, 6.90, 0.70 Hz, 1H, H 8), 7.29 (d, *J* = 8.89 Hz, 2H, H 2’), 7.05 (d, *J* = 8.89 Hz, 2H, H 3’), 7.02 (s, 1H, H 1), 6.74 (s, 1H, H 4), 4.91 (dd, *J* = 8.40, 7.40 Hz, 2H, NCH_2_CH_2_), 4.07 (q, *J* = 7.03 Hz, 2H, OCH_2_CH_3_), 3.34–3.29 (m, 2H, =NCH_2_), 2.79 (t, *J* = 6.93 Hz, 2H, CH_2_NEt_2_), 2.66 [q, *J* = 7.13 Hz, 4H, N(CH_2_CH_3_)_2_], 1.95–1.81 (m, 3H, CH_2_CHMe_2_), 2.15 (quin, *J* = 7.13 Hz, 2H, -CH_2_-), 1.42 (t, *J* = 6.98 Hz, 3H, OCH_2_CH_3_), 1.11 (d, *J* = 7.2 Hz, 6H, CHMe_2_) and 1.08 [d, *J* = 6.3 Hz, 6H, N(CH_2_CH_3_)_2_]; ^13^C NMR (101 MHz, CD_3_OD): δ 158.6 (C 4’), 153.8 (C 3), 147.5 (C 10a), 143.8 (C 2), 140.4 (C 9a), 135.3 (C 4a), 133.3 (C 1’), 133.1 (C 7), 131.3 (C 9), 130.5 (C 5a), 128.4 (C 8), 126.4 (C 3’), 117.1 (C 6), 116.9 (C 2’), 105.3 (C 1), 89.9 (C 4), 65.1 (OCH_2_CH_3_), 50.9 (CH_2_NEt_2_), 48.3 [N(CH_2_CH_3_)_2_], 47.4 (NCH_2_CH_2_), 44.9 (=NCH_2_), 38.3 (CH_2_CH), 27.6 (CHMe_2_), 25.9 (-CH_2_-), 23.1 (CHMe_2_), 15.3 (OCH_2_CH_3_) and 11.8 [N(CH_2_CH_3_)_2_]; HRMS (ESI-TOF+): *m*/*z* calculated for C_32_H_44_N_5_O: 514.3546; found: 514.3546 (MH+); IR (cm^−1^): 3381 (N-H), 2980 (Ar-C-H), 1507 (C=N), 1462 (Ar-C=C), 1253 (C-N).

*(E)-5-[2-(Dimethylamino)ethyl]-3-isobutylimino-8-methyl-2-(4-methylphenyl)amino-3,5-dihydrophenazine* (**10l**). Dark red solid (23.0 mg, 11%); *R*_f_ 0.37 [10% (*v*/*v*) methanol: chloroform]; mp. 148–150 °C (ethanol). ^1^H NMR (600 MHz, CDCl_3_ + CD_3_OD): δ 7.12 (br s, 1H, H 6), 7.08–7.04 (m, 3H, H 9, H-3’), 7.04–6.99 (m, 3H, H-7, H 2’), 6.46 (s, 1H, H 1), 5.68 (s, 1H, H 4), 3.96 (t, *J* = 7.19 Hz, 2H, NCH_2_CH_2_), 3.07 (d, *J* = 6.90 Hz, 2H, NCH_2_CH), 2.43 (t, *J* = 8.22 Hz, 2H, CH_2_NMe_2_), 2.25 (s, 6H, NMe_2_), 2.22 (s, 3H, C-8 Me), 2.18 (s, 3H, C-4’ Me), 1.92 (dquin, *J* = 13.41, 6.74 Hz, 1H, CHMe_2_), and 0.94 (d, *J* = 6.75 Hz, 6H, CHMe_2_), ^13^C NMR (151 MHz, CD_3_OD): δ 153.7 (C 3), 151.2 (C 10a), 144.8 (C 2), 138.7 (C 1’), 137.0 (C 4a), 134.6 (C 9a), 134.6 (C 5a), 134.0 (C 4’), 131.2 (C 8), 131.1 (C 3’), 129.2 (C 9), 128.7 (C 7), 122.5 (C 2’), 114.1 (C 6), 99.9 (C 1), 88.5 (C 4), 58.9 (NCH_2_CH), 54.6 (CH_2_NMe_2_), 46.0 (NMe_2_), 45.1 (NCH_2_), 31.1 (CHMe_2_), 21.6 (CHMe_2_), 21.1 (C-4’ Me) and 20.9 (C-8 Me); HRMS (ESI-TOF+): *m*/*z* calculated for C_28_H_36_N_5_: 442.2971; found: 442.2831 (MH+);IR (cm^−1^): 3221 (N-H), 2959 (Ar-C-H), 1518 (C=N) and 1464 (Ar-C=C), 1241 (C-N). 

*(E)-3-(Cyclohexylimino)-5-[2-(dimethylamino)ethyl]-8-methyl-N-p-tolyl-3,5-dihydrophenazin-2-amine* (**10m**). Maroon solid (26.0 mg, 21%); *R*_f_ 0.50 [10% (*v*/*v*) methanol:chloroform]; mp. 155–157 °C (ethanol). ^1^H NMR (400 MHz, CD_3_OD): δ 7.44 (br d, *J* = 8.80 Hz, 1H, H 6), 7.38 (br s, 1H, H 9), 7.30 (br dd, *J* = 8.80, 1.60 Hz, 3H, H 7), 7.14 (d, *J* = 8.01 Hz, 2H, H 3′), 7.08 (d, *J* = 8.60 Hz, 2H, H 2’), 6.73 (s, H 1), 1H, 6.22 (s, 1H, H 4), 4.37 (br t, *J* = 7.03 Hz, 2H, NCH_2_), 3.68–3.57 (m, 1H, =NCH), 2.60 (t, *J* = 7.60 Hz, 2H, CH_2_NMe_2_), 2.31 (s, 6H, NMe_2_), 2.29 (s, 3H, C-8 Me), 2.26 (s, 3H, C-4’ Me), 2.05–1.91, 1.85–1.73, 1.71–1.58 and 1.50–1.31 [4 × m, 10H, NCH(C_5_H_10_)]; ^13^C NMR (101 MHz, CD_3_OD): δ 152.3 (C 3), 148.8 (C 10a), 143.7 (C 2), 138.7 (C 1’), 136.9 (C 4a), 135.5 (C 9a), 134.4 (C 5a), 133.3 (C 4’), 131.3 (C 8), 131.2 (C 3’), 129.4 (C 9), 128.8 (C 7), 123.4 (C 2’), 115.3 (C 6), 103.0 (br, C 1), 89.5 (C 4), 57.1 (=NCH), 56.0 (CH_2_NMe_2_), 46.6 (NCH_2_), 46.1 (NMe_2_), 33.9 [CH(CH_2_)_2_], 26.8 and 26.2 [CH_2_(CH_2_)_2_], 21.0 (C-4’ Me) and 20.98 (C-8 Me); HRMS (ESI-TOF+): *m*/*z* calculated for C_30_H_39_N_5_: 469.3205; found: 469.3041 (MH+). IR (cm^−1^): 3231 (N-H), 2922 (Ar-C-H), 1512 (C=N) and 1460 (Ar-C=C), 1242 (C-N).

*(E)-5-[2-(Dimethylamino)ethyl]-3-(isobutylimino)-N-p-tolyl-3,5-dihydrophenazin-2-amine* (**10n**). Dark red solid (98 mg, 17% yield); R_f_ 0.50 [20% (*v*/*v*) methanol: chloroform]; mp. 140–142 °C (ethanol). ^1^H NMR (600 MHz, CD_3_OD_3_): δ 8.14 (d, *J* = 8.8 Hz, 1 H, H-6), 8.09 (d, *J* = 8.3 Hz, 1 H, H-7), 7.93 (t, *J* = 7.9 Hz, 1 H, H-9), 7.74 (t, *J* = 7.6 Hz, 1-H, H-8), 7.47 (d, *J* = 7.9 Hz, 2 H, H-3′), 7.38 (d, *J* = 7.7 Hz, 2 H, 2′), 7.37 (s, 1 H, H-1), 6.93 (s, 1 H, H-4), 5.05 (t, *J* = 7.3 Hz, 2 H, NCH_2_), 3.58 (s, 3 H, Ph-CH_3_) 3.56 (d, *J* = 7.2 Hz, 2 H, NCH_2_CH), 2.98–2.92 (m, 2 H, CH_2_NEt_2_), 2.46 (s, 2.28, 6H, CH_2_NMe_2_), (t, *J* = 13.6, 6.7 Hz, 1 H, CHMe_2_), 1.14 (d, *J* = 6.6 Hz, 6 H, CHMe_2_). ^13^C NMR (151 MHz, CD_3_OD_3_): δ 153.2 (C 3), 149.9 (C-10a), 143.8 (C 2), 137.4 (C 1’), 137.2 (C-4a), 133.7 (C-9a), 133.4 (C-5a), 130.1 (C 3), 129.8 (C 7), 129.4 (C 9), 128.8 (C 8), 124.0 (C-4’), 122.6 (C 2’), 112.9 (C 6), 100.2 (C 1), 87.6 (C 4), 56.8 (CH_2_NMe_2_), 54.6 (NCH_2_CH), 46.1 [N(CH_3_)_2_], 45.1 (C 1’), 29.6 (CHMe_2_), 21.2 (CHMe_2_), 21.1 (PhCH_3_); HRMS (ESI-TOF+): *m*/*z* calculated for C_27_H_34_N_5_: 428.2814; found: 428.2823 (M+H)+. IR (cm^−1^): 3301 (N-H), 2975 (Ar-C-H), 1510 (C=N) and 1465 (Ar-C=C), 1238 (C-N).

*(E)-5-[2-(Dimethylamino)ethyl]-3-(isobutylimino)-N-phenyl-3,5-dihydrophenazin-2-amine* (**10o***)*. Dark maroon tacky compound (6.40 mg, 25%); R_f_ 0.39 [10% (*v*/*v*) methanol: chloroform]; mp. 119–121 °C (ethanol). ^1^H NMR (400 MHz, CDCl_3_):δ 7.72 (d, *J* = 7.80 Hz, 1 H, H-6), 7.40–7.32 (m, 6 H, H-7, H-9, H-2’, H-3’), 7.07 (t, *J* = 7.22 Hz, 1 H, H 8), 6.92 (s, 1 H, H-1), 6.06 (s, 1 H, H-4), 4.29 (br s, 2 H, H 1’), 3.32 (d, *J* = 7.24 Hz, 2 H, NCH_2_CH), 2.72 (t, J = 7.80 Hz, 2-H, CH_2_NMe_2_), 2.43 [s, 6-H, N(CH_3_)_2_], 2.15 (br s, 1-H, CHMe_2_), 1.07 (d, *J* = 6.63 Hz, 6-H N(CH_2_CH_3_)_2_). ^13^C NMR (101 MHz, CDCl_3_): δ 153.1 (C 3), 150.3 (C 10a), 143.5 (C 2), 140.1 (C 1’), 137.0 (C 4a), 133.4 (C 9a), 131.1 (C 5a), 130.1 (C 7), 129.5 (C 9), 129.1 (C 3’), 129.0 (C 8), 122.2 (C 2’, C 4’), 112.8 (C 6), 100.2 (C 1), 87.6 (C 4), 57.6 (CH_2_NEt_2_), 54.5 (C 2), 46.1 [N(CH_3_)_2_], 45.0 (C 1’), 29.9 (CHMe_2_), 20.9 (CHMe_2_). HRMS (ESI-TOF+): *m*/*z* calculated for C_27_H_33_N_5_: 428.2382; found: 428.2378 (M+H)+. IR (cm^−1^): 3289 (N-H), 2950 (Ar-C-H), 1513 (C=N), 1464 (Ar-C=C), 1239 (C-N).

*(E)-3-(Cyclohexylimino)-5-[(3-dimethylamino)propyl]-N-p-tolyl-3,5-dihydrophenazin-2-amine* (**10p**). A red solid (97.4 mg, 26%); R_f_ 0.43 [20% (*v*/*v*) methanol:chloroform]; mp. 212–215 °C (ethanol). ^1^H NMR (400 MHz, CD_3_OD): δ 7.65 (d, *J* = 7.50 Hz, 2 H, H 6, H 9), 7.50 (t, *J* = 8.30 Hz, 1 H, H 7), 7.32 (t, *J* = 7.60 Hz, 1 H, H 8), 7.22 (q, *J* = 8.44 Hz, 4 H, H 2’’, H 3’’), 6.81 (s, 1 H, H 1), 6.24 (s, 1 H, H 4), 4.45–4.26 (m, 2 H, H 1’), 3.83–3.63 (m, 1 H, H 1*), 2.52 (t, *J* = 7.04 Hz, 2 H, H 3’), 2.35 (s, 3 H, PhCH_3_), 2.31 [s, 6 H, N(CH_3_)_2_], 2.07–1.94 (m, 4 H, H 2’, H 2*), 1.90 (br s, 2 H, H 2*), 1.77 (br d, *J* = 12.7Hz, 2 H, H 4*), 1.61–1.45 (m, 4 H, H 3*). ^13^C NMR (151 MHz, CD_3_OD): δ 152.6 (C 3), 150.3 (C 10a), 145.0 (C 2), 138.6 (C 1’’), 138.0 (C 4a), 135.4 (C 9a), 134.7 (C 5a), 131.3 (C 3’’), 131.2 (C 7), 130.8 (C 4’’), 129.6 (C 9), 125.6 (C 8), 123.4 (C 2’’), 115.3 (C 6), 101.7 (C 1), 89.3 (C 4), 65.6 (C 1*), 57.7 (C 3’), 46.0 [N(CH_3_)_2_], 45.7 (C 1’), 34.4 (C 2*), 27.0 (C 4*), 26.3 (C 2’), 25.2 (C 3*), 21.1 (PhCH_3_). HRMS (ESI-TOF+): *m*/*z* calculated for C_30_H_38_N_5_: 468.3726; found: 468.3102 (M+H)+. IR (cm^−1^): 3253 (N-H), 2922 (Ar-C-H), 1514 (C=N), 1459 (Ar-C=C), 1210 (C-N).

### 3.2. Method for Cyclic Voltammetry

Cyclic voltammetry was carried out using a BAS-100W electrochemical analyzer using a one-compartment three-electrode system composed of an Ag/Ag^+^ reference electrode (0.01 M AgNO_3_), a platinum wire as the auxiliary electrode, and a platinum disc as the working electrode. The supporting electrolyte solution was 0.1 M aqueous tetrabutylammonium perchlorate in dried acetonitrile (100 mL). All measurements were carried out at 1, 3, or 5 μM of the riminophenazines, at a scan rate of 100 mV.s^−1^, unless otherwise stated. All analyses were run in duplicate under an atmosphere of nitrogen at room temperature. The solutions were deoxygenated by purging under a stream of nitrogen through the solution for 4 min prior each run. The platinum disc electrode was polished after every run.

### 3.3. Antitubercular Testing (MIC_99_ Determination) Method

A working stock solution of 10 µM of the test compound in fresh DMSO was prepared and stored at −70 °C until used. All compounds were tested at 5, 3, and 1 µM point concentrations for the initial screens. Mycobacterium tuberculosis H_37_Rv inocula were prepared from cultures grown on Lowenstein Jensen (LJ) slants. Mycobacterial suspensions were prepared in saline, and the turbidity was adjusted to 0.5 McFarland units. Aliquots of 100 µL were inoculated into MGIT tubes and incubated at 37 °C until the inoculum became positive (about three days). Then, 0.5 mL of positive MGIT cultures were transferred into MGIT tubes containing the test compounds. Controls consisting of a 1:100 dilution of the positive MGIT culture control in saline, 1.2% DMSO control in saline (concentration equivalent to that used when introducing the drug substance), and a 0.05 µg/mL isoniazid (INH) positive control in saline were also prepared. For mycobacterial growth evaluation, the MGIT 960 system (Becton Dickinson, Sparks, MD, USA) was used, where *M. tuberculosis* growth is observed through fluorescent changes due to oxygen consumption during mycobacterial growth. Incubation at 37 °C was continued in the MGIT system, and the growth units (GU) were recorded hourly. For MIC_99_ evaluations, the 1% bacterial control culture was used; the MIC_99_ of the compound was determined relative to the growth units of the control (GU_400). When the GU of the control reached 400, the results were interpreted. If the drug-containing tube showed GU > 400, the material was defined as inducing resistance; if it showed GU ≤ 400, susceptibility of the mycobacteria to the drug substance was indicated at that concentration of drug substance. Compounds that showed GU = 0 at 1 µM were considered to have an MIC ≤ 1 µM, and further MIC determinations on these materials using concentrations ranging from 0.0625–1 µM were performed. All experiments were conducted in triplicate, with three independent experiments performed each time and the averages are reported. Statistically, the percentage range of standard deviation from mean showed the two concentrations (3 and 5 µM) having a close range as compared to the lowest concentration (see Supplementary data).

### 3.4. Protocol for Toxicity Evaluation (IC_50_) Method

The WI-38 cell line—normal Human Fetal Lung Fibroblast from ECACC was routinely maintained as a monolayer cell culture at 37 °C, 5% CO_2_, 95% air, and 100% relative humidity in EMEM containing 10% fetal bovine serum, 2 mM L-glutamine, and 50 µg/mL gentamicin. For screening experiment, the cells (21–50 passages) were inoculated in 96-well microtiter plates at plating densities of 10,000 cells/well and were incubated for 24 h. After 24 h, the cells were treated with the experimental drugs, which were previously dissolved in DMSO and diluted in medium to produce five concentrations. The process was performed in triplicate and the data presented are means of three repeats performed in triplicate. Cells without drug addition served as control. The blank contains complete medium without cells. Parthenolide was used as a standard. The plates were incubated for 48 h after addition of the compounds. Viable cells were fixed to the bottom of each well with cold 50% trichloroacetic acid, washed, dried, and dyed by SRB. Unbound dye was removed, and protein-bound dye was extracted with a 10 mM Tris base for optical density determination at the wavelength 540 nm using a multiwell spectrophotometer. Data analysis was performed using GraphPad Prism software. 50% of cell growth inhibition (IC_50_) was determined by non-linear regression.

## 4. Conclusions

A series of variously substituted phenazines having ionizable aminoalkyl chains installed at N-5 of the riminophenazine system as part of the strategy to develop less lipophilic compounds with high anti-TB activity are reported. While it is known that lipophilicity is an important determinant of antimycobacterial activity as well as pigment deposition in fatty tissues [14], the inclusion of aminoalkyl sidechains has generated reasonably active materials with the potential to further enhance activity while still providing a “handle” for improved compounds. Remarkably, compounds with methyl groups at both R_3_ and R_4_ positions with Me_2_N(CH_2_)_2_- at N-5 position (**10l** and **10m**) demonstrated both lower ClogP values and were also potent against *M. tuberculosis*. The data obtained give a preliminary indication that the more commonly used N-5 phenyl moieties may be substituted without abrogating activity. Future work will focus on refining this further, followed by pharmacokinetic testing.

## Figures and Tables

**Table 1:** Structure–activity assessment of riminophenazines **10**.

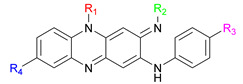							
Compound	R_1_	R_2_	R_3_	R_4_	LogP ^1^	IC_50_ (µM) ^2^	MIC_99_ (µM) ^3^
* Clofazimine **10b** **10o**	4-Clphenyl Et_2_N(CH_2_)_3_- Me_2_N(CH_2_)_2_-	iPr iBu iBu	Cl H H	H H H	7.50 4.21 3.43	68.62 4.44 2.45	<1 3 3
**10d**	Me_2_N(CH_2_)_3_-	iBu	Me	H	4.02	2.47	3
**10e**	Et_2_N(CH_2_)_3_-	iBu	Me	H	4.70	1.63	5
**10l** **10n**	Me_2_N(CH_2_)_2_- Me_2_N(CH_2_)_2_-	iBu iBu	Me Me	Me H	4.73 3.91	2.86 3.89	<1 3
**10g**	Me_2_N(CH_2_)_2_-	iBu	OEt	H	3.64	12.30	5
**10i**	Me_2_N(CH_2_)_3_-	iBu	OEt	H	3.74	4.81	5
**10j**	Et_2_N(CH_2_)_3_-	iBu	OEt	H	4.42	5.35	3
**10k**	Et_2_N(CH_2_)_3_-	iPent	OEt	H	4.77	3.81	5
**10a**	Me_2_N(CH_2_)_2_-	cHex	H	H	3.75	9.01	3
**10c**	Me_2_N(CH_2_)_2_-	cHex	Me	H	4.31	5.99	5
**10p**	Me_2_N(CH_2_)_3_-	cHex	Me	H	4.34	5.08	<1
**10f**	Et_2_N(CH_2_)_3_-	cHex	Me	H	5.02	4.37	3
**10m**	Me_2_N(CH_2_)_2_-	cHex	Me	Me	4.41	3.64	<1
**10h**	Me_2_N(CH_2_)_2_-	cHex	OEt	H	3.96	11.10	5
**INH**							<1

^1^ LogP calculated using ChemDraw version 12.0; ^2^ Cytotoxicity against rapidly dividing human fibroblasts (WI38 cell line); ^3^ MIC_99_: lowest concentration of the material inhibiting 99% of *M. tuberculosis* H_37_Rv growth in culture. * Data taken from literature [26,27], INH = Isoniazid.

**Table 2:** Redox potentials (V vs. Ag/Ag+) of selected riminophenazines.

Compound	*E*_pa_/V	*E*_pc_/V	*E*_½_/V	Δ*E*
**10b**	–0.0392	–0.41	–0.22	0.37
**10e**	–0.215	–0.971	–0.59	0.76
**10i**	–0.0218	–0.28	–0.15	0.25
**10n**	–0.0021	–0.26	–0.13	0.26
**10j**	–0.077	–0.38	–0.23	0.30
**10c**	–0.0055	–0.49	–0.24	0.35
**10f**	–0.0606	–0.41	–0.24	0.35
**10m**	–0.0305	–0.37	–0.20	0.34

Measured in CH_3_CN containing 0.1 M [n-Bu_4_N][ClO_4_] at a scan rate of 100 mV s^−1^ and referenced to Ag/Ag+. *E*_pa_ = anodic potential peak. *E*_pc_ = cathodic potential peak. *E*_½_ = Half wave potential [*E*_½_ = (*E*_pa_ + *E*_pc_)/2]. Δ*E* = *E*_pa_ − *E*_pc_.

## Data Availability

Not applicable.

## Supplementary Materials

The following are available online, ^1^H and ^13^C NMR spectra of the synthesized target compounds.
