# Supplementary material for: Riminophenazine Derivatives as Potential Antituberculosis Agents: Synthesis, Biological, and Electrochemical Evaluations
Source: Molecules. 2021 Jul 10;26(14):4200. doi: 10.3390/molecules26144200 (PMC8303532; doi:10.3390/molecules26144200)
Supplement: Supplementary file 1 [file molecules-26-04200-s001.zip › molecules-1202343-supplementary.pdf]

Supporting Information

Article

Riminophenazine derivatives as potential antituberculosis agents: synthesis, biological and electrochemical evaluations

Mpelegeng V. Bvumbi [1,2,3,*,†], Chris van der Westhuyzen [2,†], Edwin M. Mmutlane [1] and Andile H. Ngwane [4]

[1] Department of Chemistry, University of Johannesburg, Auckland Park 2006, South Africa; edwinm@uj.ac.za
[2] CSIR Biosciences, Pretoria 0001, South Africa; cwvdwesthuyzen@csir.co.za
[3] Department of Chemistry, University of Venda, Private X5050, Thohoyandou 0950, South Africa
[4] Division of Molecular Biology and Human Genetics, Department of Biomedical Sciences, Faculty of Medicine and Health Sciences, Stellenbosch University, Tygerberg 7505, South Africa; andilengwane@gmail.com
* Correspondence: mpelegeng.bvumbi@univen.ac.za; Tel.: +27-15-962-8756
† These authors contributed equally to this work.

1. Materials and Methods

The reactions were monitored for their completion by thin layer chromatography (TLC) using aluminium-backed Macherey-Nagel ALUGRAM Sil G/UV254 plates pre-coated with 0.25 mm silica gel 60Å. Column chromatography was carried out on silica gel 60 (particle size 200-300 mesh) with a silica to compound ratio of 30:1 by mass or neutral alumina as the adsorbent for conventional preparative chromatography.

NMR spectra were recorded on a Varian INOVA 400 MHz system or a Varian PremiumShield VNMRS 600 MHz system operating at 399.94 MHz or 599.74 MHz, respectively. Chemical shifts (δ) are reported in parts per million (ppm) relative to tetramethylsilane (TMS) or against the residual protonated solvent signal as reference. Coupling constants (J) are reported in Hz.

Melting points were obtained on a Reichert Hot Stage or a Mettler FP62 melting point apparatus, and are uncorrected. Low and high resolution mass spectra were recorded on a Waters Acquity UPLC coupled in tandem to a Waters photodiode array (PDA) detector and a SYNAPT G1 HDMS mass spectrometer.

*1.1 Synthesis of compounds **2a-g***

Method A: A mixture of the required 1-fluoro-2-nitrobenzene **1a** and alkyldiamine (1.5 eq.) in isopropanol (1.2 M) was heated at 90-100 °C for 16h. The mixture was cooled, extracted from water with ethyl acetate. The organic extracts were dried (MgSO4), filtered through celite and concentrated in vacuo, and the residue purified as indicated.

Method B: The required 1-chloro-2-nitrobenzene **1b,c** and alkyldiamine (2 eq.), or alkyldiamine (0.91 eq.) with potassium carbonate (2 eq.), as a mixture in isopropanol (1 M) or anhydrous dimethylformamide (2.3 M) was heated at 90-100 °C for 24h. The mixture was cooled, filtered and concentrated. Extraction from water with dichloromethane was followed by back-extraction of the DCM isolates with 6 M aqueous HCl. The aqueous phase was washed with DCM, basified with sodium hydroxide pellets and re-extracted with DCM. The organic extracts were dried ($MgSO_4$), filtered through celite and concentrated in vacuo, and the residue purified as indicated.

N^1,N^1-dimethyl-N^2-(2-nitrophenyl)ethane-1,2-diamine **(2a)**. Orange oil, (9.39 g, 74%); R_f 0.37 [40% (v/v) methanol: ethyl acetate]. ^{1}H NMR (400 MHz, $CDCl_3$): δ 8.30 (br s, 1H, NH), 8.17 (dd, *J* = 8.6, 1.2 Hz, 1H, H 3), 7.43 (ddd, *J* = 7.4, 6.8, 1.2 Hz, 1H, H 5), 6.84 (d, *J* = 8.6 Hz, 1H, H 6), 6.64 (ddd, *J* = 7.4, 6.9, 0.8 Hz,

1H, H 4), 3.37 (t, *J* = 6.3 Hz, 2H, $NHCH_2$-), 2.65 (dt, *J* = 6.2, 5.1 Hz, 2H, -CH_2NMe_2), 2.29 [s, 6H, N(CH_3)2]; ^{13}C NMR (101 MHz, $CDCl_3$): δ 145.3 (C 1), 136.1 (C 5), 132.0 (C 2), 126.9 (C 3), 115.1 (C 4), 113.9 (C 6), 57.4 (-CH_2NMe_2), 45.2 [N(CH_3)$_2$], 40.7 ($NHCH_2$-); HRMS (ESI-TOF+): m/z calculated for $C_{10}H_{16}N_3O_2$: 210.1243; found: 210.1267 (MH+).

N^1,N^1-dimethyl-N^3-(2-nitrophenyl)propane-1,3-diamine **(2b)**. Orange oil (5.73 g, 64%); *R_f* 0.18 [50% (v/v) methanol:ethyl acetate]. ^{1}H NMR (400 MHz, $CDCl_3$) δ 8.42 (br s, 1H, NH), 8.17 (dd, *J* = 8.6, 1.6 Hz, 1H, H 3), 7.42 (ddd, *J* = 7.0, 1.2 Hz, 1H, H 5), 6.88 (d, *J* = 8.6 Hz, 1H, H 6), 6.63 (ddd, *J* = 8.5, 7.1, 1.2 Hz, 1H, H 4), 3.39 (dt, *J* = 6.6, 5.5 Hz, 2H, $NHCH_2$-), 2.45 (t, *J* = 6.6 Hz, 2H, -CH_2NMe_2), 2.28 [s, 6H, N(CH_3)$_2$], 1.89 (quin, *J* = 6.7 Hz, 2H, -CH_2-); ^{13}C NMR (101 MHz, $CDCl_3$): δ 145.6 (C 1), 136.1 (C 5), 131.9 (C 2), 126.9 (C 3), 114.9 (C 4), 113.7 (C 6), 57.4 (-CH_2NMe_2), 45.4 [N(CH_3)$_2$], 41.7 ($NHCH_2$), 26.6 (-CH_2-); HRMS (ESI-TOF+): m/z calculated for $C_{11}H_{18}N_3O_2$: 224.1399; found: 224.1407 (MH+).(1)

N^1,N^1-diethyl-N^3-(2-nitrophenyl)propane-1,3-diamine (**2c).** Dark orange oil (11.3, g 63%); *R_f* 0.25 [50% (v/v) methanol:ethyl acetate]. ^{1}H NMR (400 MHz, $CDCl_3$) δ 8.37 (br s, 1H, NH), 8.09 (br d, *J*= 8.6 Hz, 1H, H 3), 7.36 (br t, *J* = 7.8 Hz, 1H, H 5), 6.81 (d, *J* = 8.6 Hz, 1H, H 6), 6.55 (ddd, *J* = 7.4, 2.2 Hz, 1H, H 4), 3.31 (dt, *J* = 6.1, 5.1 Hz, 2H, $NHCH_2$-), 2.49 (m, 6H, -CH_2NMe_2, N(CH_2Me)$_2$], 1.80 (quin, *J* = 6.6 Hz, 2H, -CH_2-), 0.98 [t, *J* = 7.1 Hz, 6H, N(CH_2Me)$_2$]; ^{13}C NMR (101 MHz, $CDCl_3$): δ 145.3 (C 1), 135.9 (C 5), 131.4 (C 2), 126.6 (C 3), 114.6 (C 4), 113.6 (C 6), 50.5 (-CH_2NEt_2), 46.7 [N(CH_2Me)$_2$], 41.8 ($NHCH_2$-), 26.2 (-CH_2-), 11.4 [N(CH_2Me)$_2$]; HRMS (ESI-TOF+): m/z calculated for $C_{11}H_{18}N_3O_2$: 224.1399; found: 224.1407 (MH+).[1]

N^1,N^1-dimethyl-N^2-(4-methyl-2-nitrophenyl)ethane-1,2-diamine **(2d).** Orange oil (4.12 g, (52%); *R_f* 0.33 [5% (v/v) methanol: chloroform]. ^{1}H NMR (400 MHz, $CDCl_3$): δ 8.16 (s, 1H, NH), 7.95 (d, *J* = 1.2 Hz, 1H, H 3), 7.25 (dd, *J* = 8.8, 2.2 Hz, 1H, H 5), 6.75 (d, *J* = 8.6 Hz, 1H, H 6), 3.33 (dt, *J* = 6.3, 5.1 Hz, 2H, $NHCH_2$-), 2.61 (t, *J* = 6.4 Hz, 2H, -CH_2NMe_2), 2.30 [s, 6H, N(CH_3)$_2$], 2.25 (s, 3H, C4-CH_3); ^{13}C NMR (101 MHz, $CDCl_3$): δ 143.6 (C 1), 137.5 (C 5), 131.5 (C 2), 126.0 (C 3), 124.5 (C 4), 113.8 (C 6), 57.5 (-CH_2NMe_2), 45.2 [N(CH_3)$_2$], 40.8 ($NHCH_2$-), 19.8 (C4-CH_3); HRMS (ESI-TOF+): m/z calculated for $C_{11}H_{18}N_3O_2$: 224.1399; found: 224.1378 (MH+).

N^1,N^1-dimethyl-N^3-(4-methyl-2-nitrophenyl)propane-1,3-diamine **(2e).** Orange oil (10.2 g, 72%); *R_f* 0.30 [5% (v/v) methanol:chloroform]. ^{1}H NMR (400 MHz, $CDCl_3$) δ 8.18 (br s, 1H, NH), 7.88 (s, 1H, H 3), 7.18 (dd, *J* 8.8, 1.7 Hz, 1H, H 5), 6.72 (d, *J* 8.8 Hz, 1H, H 6), 3.30 (dd, *J* 12.2, 6.6 Hz, 2H, $NHCH_2$-), 2.38 (t, *J* 6.8 Hz, 2H, -CH_2NMe_2), 2.21 [s, 6H, N(CH_3)$_2$], 2.18 (s, 3H, C4-CH_3), 1.81 (quin, *J* 6.8 Hz, 2H, -CH_2-); ^{13}C NMR (101 MHz, $CDCl_3$): δ 143.7 (C 1), 137.5 (C 5), 131.2 (C 2), 125.8 (C 3), 124.3 (C 4), 113.4 (C 6), 57.2 (-CH_2NMe_2), 45.2 [N(CH_3)$_2$], 41.5 ($NHCH_2$-), 26.5 (-CH_2-), 19.7 (C-4 CH_3); HRMS (ESI-TOF+): m/z calculated for $C_{12}H20N_3O_2$: 238.1556; found: 238.1581 (MH+).[2]

N^1-(4-chloro-2-nitrophenyl)-N^2,N^2-dimethylethane-1,2-diamine **(2f)**. Orange solid (7.04 g, 53%); *R_f* 0.29 [30% (v/v) ethyl acetate:hexane]. ^{1}H NMR (400 MHz, $CDCl_3$): δ 8.28 (1H, s, NH), 8.13 (d, *J* = 2.5 Hz, 1H, H 3), 7.34 (ddd, *J* = 9.2, 2.5, 0.6 Hz, 1H, H 5), 6.77 (d, *J* = 9.2 Hz, 1H, H 6), 3.31 (dd, *J* = 10.9, 6.2 Hz, 2H, $NHCH_2$-), 2.61 (t, *J* = 6.2 Hz, 2H, -CH_2NMe_2), 2.28 [s, 6H, N(CH_3)$_2$]; ^{13}C NMR (101 MHz, $CDCl_3$): δ 143.9 (C 1), 136.2 (C 5), 131.8 (C 2), 125.9 (C 3), 119.8 (C 4), 115.3 (C 6), 57.2 (-CH_2NMe_2), 45.2 [N(CH_3)$_2$], 40.8 ($NHCH_2$-); HRMS (ESI-TOF+): m/z calculated for $C_{10}H_{15}N_3O_2Cl$: 244.0853; found: 244.0876 (MH+).

N^1-(4-chloro-2-nitrophenyl)-N^3,N^3-dimethylpropane-1,3-diamine **(2g).** Red oil (5.13 g, 72%); *R_f* 0.27 [10% (v/v) methanol:chloroform]. ^{1}H NMR (400MHz, $CDCl_3$) δ 8.48 (br s, 1H, NH), 8.12 (d, *J* = 2.5 Hz, 1H, H 3), 7.33 (dd, *J* = 9.2, 2.5 Hz, 1H, H 5), 6.81 (d, *J* = 9.2 Hz, 1 H, H 6), 3.34 (dd, *J* = 11.8, 6.6 Hz, 2H, NHCH2-), 2.41 (t, *J* = 6.5Hz, 2H, -CH_2NMe_2), 2.24 [s, 6H, N(CH_3)$_2$], 1.84 (quin, *J* = 6.6 Hz, 2H, -CH_2-); ^{13}C NMR (101 MHz, $CDCl_3$): δ 144.2 (C 1), 136.2 (C 5), 131.7 (C 2), 125.9 (C 3), 119.6 (C 4), 115.1 (C 6), 57.4 (-CH_2NMe_2), 45.4 [N(CH_3)$_2$], 42.1 ($NHCH_2$-), 26.4 (-CH_2-); HRMS (ESI-TOF+): m/z calculated for $C_{11}H_{17}N_3O_2Cl$: 258.1009; found: 258.1030 (MH+).

*1.2 Synthesis of compounds **3a-e***

Method A: Compound **2** (1 eq.) in ethanol (0.6 M) was mixed with 10% palladium on carbon (0.5% m/m) and hydrazine hydrate (5 eq.) to give crude products. The filter bed was washed with ethanol and the combined filtrates concentrated in vacuo to afford the desired products. No further purification was performed.

Method B: Compound **2** (1 eq.) in ethanol (0.6 M) was mixed with 5% palladium on carbon (0.5% m/m) and sealed in an autoclave under 2.7 atm hydrogen gas pressure at room temperature for 2.5h to give crude products. No further purification was performed.

N^1-[2-(Dimethylamino)ethyl]-1,2-phenylenediamine **(3a)**. Pale yellow wax (4.29 g, 79%); R_f 0.29 [5% (v/v) 25% ammonia: acetone]. ^{1}H NMR (400 MHz, $CDCl_3$): δ 6.83 (td, *J* = 7.8, 2.3 Hz, 1H, H-5), 6.74 – 6.67 (m, H-3, 2H, H-4), 6.68 – 6.65 (m, 1H, H-6), 3.39 and 3.33 (2 x br s, NH, 3H, NH_2), 3.17 (t, *J* = 6.1 Hz, 2H, $NHCH_2$-), 2.60 (t, *J* = 5.1 Hz, 2H, -CH_2NMe_2), 2.27 [s, 6H, $N(CH_3)_2$]; ^{13}C NMR (101 MHz, $CDCl_3$): δ 137.7 (C 1), 134.5 (C 2), 120.3 (C 5), 118.4 (C 4), 116.0 (C 3), 111.7 (C 6), 58.2 (-CH_2N), 45.2 (NMe_2), 41.5 ($NHCH_2$-).

N^1-[3-(Dimethylamino)propyl]-1,2-phenylenediamine **(3b).** Pale yellow oil (7.29 g, 80%); R_f 0.28 [50% (v/v) methanol:ethyl acetate].; ^{1}H NMR (400 MHz, $CDCl_3$): δ 6.80 (td, *J* = 7.4, 1.6Hz, 1H, H-5), 6.71 – 6.64 (m, 2H, H-3, H-4), 6.65 – 6.62 (m, 1H, H-6), 3.37 and 2.95 (2 x br s, 3H, NH, NH_2), 3.16 (t, *J* = 6.6 Hz, 2H, $NHCH_2$-), 2.42 (t, *J* = 6.8 Hz, 2H, - CH_2NMe_2), 2.25 [s, 6H, $N(CH_3)_2$], 1.83 (quin, *J* = 6.7 Hz, 2H, -CH_2-); ^{13}C NMR (101 MHz, CDCl3): δ 137.8 (C 1), 134.0 (C 2), 120.3 (C 5), 118.1 (C 4), 116.0 (C 3), 111.3 (C 6), 58.2 (-CH_2NMe_2), 45.4 (NMe_2), 43.2 ($NHCH_2$-), 27.0 (-CH_2-).

N^1-[3-(Diethylamino)propyl]-1,2-phenylenediamine (**3c**). Viscous maroon oil (3.94 g, 43%); R_f 0.29 [10% (v/v) methanol:ethyl acetate]. ^{1}H NMR (400 MHz, $CDCl_3$): δ 6.81 (td, *J* = 7.0, 1.6 Hz, 1H, H-5), 6.73 – 6.66 (m, 2H, H-3, H-4), 6.66 – 6.63 (m, 1H, H-6), 3.42 (br s, 3H, NH, NH_2), 3.19 (t, *J* = 6.4 Hz, 2H, $NHCH_2$-), 2.62 (t, *J* = 6.6 Hz, 2H, -CH_2N-), 2.60 [q, *J* = 7.0 Hz, 4H, $N(CH_2Me)_2$], 1.86 (quin, *J* = 6.6 Hz, 2H, -CH_2-), 1.07 [t, *J* = 7.0 Hz, 6H, $N(CH_2Me)_2$]; ^{13}C NMR (101 MHz, $CDCl_3$): δ 137.9 (C 1), 134.2 (C 2), 120.3 (C 5), 118.1 (C 4), 115.9 (C 3), 111.3 (C 6), 52.0 (-CH_2N), 46.9 [$N(CH_2Me)_2$], 43.8 ($NHCH_2$-), 26.4 (-CH_2-), 11.5 [$N(CH_2Me)_2$].

N^1-[2-(Dimethylamino)ethyl]-4-methyl-1,2-phenylenediamine **(3d).** Viscous maroon oil (3.06 g, 87%); R_f 0.26 [5% (v/v) methanol:chloroform]. ^{1}H NMR (400 MHz, $CDCl_3$): δ 6.61 (dd, *J* = 7.8, 2.0, 1H, H-6), 6.59 (d, *J* = 7.8, 1H, H-5), 6.55 (d, *J* = 2.0, 1H, H-3), 3.43 (br s, NH, 3H, NH_2), 3.15 (dd, *J* = 6.4, 5.5 Hz, 2H, $NHCH_2$-), 2.60 (dd, *J* = 6.4, 5.5 Hz, 2H, -CH_2NMe_2), 2.28 (s, 6H, NMe_2), 2.24 (s, 3H, C-4 CH_3); ^{13}C NMR (101 MHz, $CDCl_3$): δ 135.1 (C 1), 135.1 (C 2), 128.3 (C 4), 120.4 (C 5), 116.9 (C 3), 112.6 (C 6), 58.3 (-CH_2NMe_2), 45.2 (NMe_2), 42.1 ($NHCH_2$-), 20.6 (C4-CH_3).

N^1-[3-(Dimethylamino)propyl]-4-methyl-1,2-phenylenediamine **(3e).**Fluffy dark purple solid (7.84 g, 90%); R_f 0.26 [50% (v/v) methanol:chloroform]. ^{1}H NMR (400 MHz, $CDCl_3$): δ 6.59 – 6.49 (m, H-3, H-5, 3H, H-6), 3.42 (br s, 3H, NH, NH_2), 3.11 (t, *J* = 6.8 Hz, 2H, $NHCH_2$-), 2.42 (t, *J* = 7.0 Hz, 2H, -CH_2NMe_2), 2.24 [s, 6H, $N(CH_3)_2$], 2.19 (s, 3H, C-4 CH_3), 1.80 (quin, 2H, *J* = 6.8 Hz, -CH_2-); ^{13}C NMR (101 MHz, $CDCl_3$): δ 135.1 (C 1), 134.6 (C 2), 127.9 (C 4), 120.3 (C 5), 116.9 (C 3), 112.1 (C 6), 58.0 (-CH_2NMe_2), 45.2 (NMe_2), 43.4 ($NHCH_2$-), 27.0 (-CH_2-), 20.6 (C4-CH_3); HRMS (ESI-TOF+): m/z calculated for $C_{12}H_{22}N_3$: 208.1814; found: 208.1855 (MH+).

*1.3 Synthesis of compounds **6a-c***

The chosen aniline **5a-d** (1 eq.) and 1,5-difluoro-2,4-dinitrobenzene **4** (1 eq.) in 1,4-dioxane was treated with triethylamine (1 eq.) dropwise, then the solution was stirred at 70-80°C for 4h. After cooling to room temperature, the reactions were partitioned between water and ethyl acetate. The combined organic layers were dried ($MgSO_4$) and the solvent was removed in vacuo to give crude solids, which were purified by recrystallization from ethyl acetate/hexane.

N-Phenyl-2,4-dinitro-5-fluoroaniline (**6a**). Yellow crystals (4.20 g, 77%); R_f 0.32 [20% (v/v) ethyl acetate: hexane]; mp. 163.3-165.3 °C (ethyl acetate and hexane mixtures). ^{1}H NMR (400 MHz, DMSO-d$_6$): δ 9.96 (s, 1H, NH), 9.16 (d, J_{HF} = 7.93 Hz, 1H, H 3), 7.53 (m, 2H, H 2'), 7.43 (m, 1H, H 4'), 7.31 (m, 2H, H 3'), 6.82 (d, J_{HF} = 13.1 Hz, 1H, H 6); ^{13}C NMR (101 MHz, DMSO-d$_6$): δ 159.7 (d, J_{CF} = 271.4 Hz, C 5), 148.3 and 148.2 (2 x s, C-2 and C 4), 136.2 (C 1'), 130.5 (C 2'), 128.2 (C 4'), 125.6 (C 3'), 127.8 (C 3), 127.3 (C 1), 103.2 (d, J_{CF} = 27.8 Hz, C 6); HRMS (ESI-TOF+) calculated for $C_{12}H_7N_3O_4F$: 276.0421, found 276.0434 (MH+); IR (cm-1): 3305 (N-H stretch), 1573 (C=C stretch), 1513 (NO_2), 1048 (C-F stretch).

N-(4-Methylphenyl)-2,4-dinitro-5-fluoroaniline (**6b**). Bright orange crystals (4.76 g, 83%); R_f 0.33 [20% (v/v) ethyl acetate: hexane]; mp. 172.3-174.3 °C (ethyl acetate/hexane). ^{1}H NMR (400 MHz, DMSO-d$_6$): δ 9.89 (s, 1H, NH), 9.16 (d, 1H, J_{HF} = 8.85 Hz, H 3), 7.31 (m, 2H, H 3'), 7.18 (m, 2H, H 2'), 6.77 (, J_{HF} = 13.4 Hz, 1H, d H 6), 2.43 (s, 1H, 3H, C 4' CH_3); ^{13}C NMR (101 MHz, DMSO-d$_6$): δ 159.7 (d, J_{CF} = 267.0 Hz, C 5), 148.1 and 148.0 (2 x s, C-2 and C 4), 136.9 (C 4'), 134.6 (C 1'), 130.3 (C 3'), 127.9 (C 1), 127.3 (C 3), 103.0 (d, J_{CF} = 27.4 Hz, C 6), 20.6 (C 4' CH_3); HRMS (ESI-TOF+) m/z calculated for $C_{13}H_9N_3O_4F$: 290.0577, found 290.0597 (M - H); IR (cm-1): 3317 (N-H stretch), 1576 (C=C stretch), 1517 (NO_2), 1046 (C-F stretch).

N-(4-Ethoxyphenyl)-2,4-dinitro-5-fluoroaniline (**6c**). Bright red crystals (3.55 g, 71%); R_f 0.39 [20% (v/v) ethyl acetate:hexane]; mp. 129-131 °C (ethyl acetate and hexane mixtures). ^{1}H NMR (400 MHz DMSO-d$_6$): δ 9.82 (s, 1H, NH), 9.16 (d, J_{HF} = 7.3 Hz, 1H, H 3), 7.19 (m, 2H, H 3'), 7.01 (m, 2H, H 2'), 6.68 (d, J_{HF} = 13.4 Hz, 1H, H 6), 4.09 (q, J = 6.9 Hz, 2H, OCH_2CH_3), 1.46 (t, J = 6.9 Hz, 3H, OCH_2CH_3); ^{13}C NMR (101 MHz, $CDCl_3$): δ 159.3 (d, J_{CF} = 277.0 Hz, C 5), 158.8 (C 4'), 149.2 and 149.1 (2 x s, C-2 and C 4), 128.5 (C 1'), 127.8 (C 3), 127.4 (C 3'), 127.5 (C 1), 116.1 (C 2'), 103.0 (d, J_{CF} = 27.8 Hz, C 6), 63.9 (CH_2CH_3), 14.7 (CH_2CH_3); HRMS (ESI-TOF) calculated for $C_{14}H_{11}N_3O_5F$: 320.0683, found 320.0710 (MH+); IR (cm-1): 3302 (N-H stretch), 1578 (C=C stretch), 1506 (NO_2), 1048 (C-F stretch).

*1.4 Synthesis of compounds **7a-k***

The chosen phenylenediamine **3** (1.5 eq.), selected dinitroaniline **6** (1 eq.), *N,N*-diisopropylethylamine (1 eq.) and dry ethanol (0.2M) were allowed to allowed to react under reflux for 16h and cooled to room temperature. The reactions were partitioned between water and ethyl acetate, dried ($MgSO_4$) and purified by recrystallization.

N^1-[2-(2-Dimethylaminoethyl)aminophenyl]-N^3-phenyl-4,6-dinitro-1,3-phenylenediamine (**7a**). Fine bright orange solid (3.06 g, 72%); R_f 0.58 [10% (v/v) methanol:chloroform]; mp. 171.3-173.3 °C (methanol/chloroform). ^{1}H NMR (400 MHz, $CDCl_3$): δ 9.74 (br s, 1H, C3-NH), 9.32 (s, 1H, H 5), 9.26 (br s, 1H, C1-NH), 7.30- 7.23 (m, 2H, H 3''), 7.18 – 7.11 (m, 2H, H 4′, H 5′), 7.11 – 7.07 (m, 2H, H 2''), 7.03 (dd, J = 7.70, 6.10 Hz, 1H, H 4''), 6.69-6.63 (m, 2H, H 3′, H 6′), 6.22 (s, 1H, H 2), 4.42 (br. t, J = 4.5 Hz, 1H, $NHCH_2$), 3.10 (q, J = 6.0 Hz, 2H, $NHCH_2$), 2.46 (t, J = 6.3 Hz, 2H, CH_2NMe), 2.15 [s, 6H, $N(CH_3)_2$]; ^{13}C NMR (101 MHz, $CDCl_3$): δ 147.7 and 146.4 (C 4 and C 6), 144.1 (C 1′), 137.3 (C 1''), 129.5 (C 5), 129.4 (C 3''), 129.1 (C 6′), 129.0 (C 4''), 126.2 (C 4′), 125.5 and 125.4 (C-1 and C 3), 124.0 (C 2''), 122.3 (C 2′), 117.1 (C 6′), 111.9 (C 3′), 96.0 (C 2), 57.8 (CH_2NMe), 45.2 [$N(CH_3)2$], 41.1 ($NHCH_2$); HRMS (ESI-TOF+): m/z calculated for $C_{22}H_{25}N_6O_4$: 437.1937; found: 437.1942 (MH+).

N^1-[2-(3-Dimethylaminopropyl)aminophenyl]-N^3-phenyl-4,6-dinitro-1,3-phenylenediamine (**7b**). Bright maroon crystals (2.43 g, 75%); R_f 0.38 [50% (v/v) methanol: ethyl acetate]; mp. 194.8-196.8 °C (ethanol). ^{1}H NMR (400 MHz, $CDCl_3$): δ 9.75 (br s, 1H, C3-NH), 9.33 (s, 1H, H 5), 9.26 (br s, 1H, C1-NH), 7.30 –

7.23 (m, 2H, H 3''), 7.18 – 7.01 (m, 4H, H 2'', H 4', H 5′), 6.97 (br dd, *J* = 8.0, 1.6 Hz, 1H, H 4''), 6.64 – 6.58 (m, 2H, H 3′, H 6′), 6.18 (s, 1H, H 2), 5.63 (s, 1H, $NHCH_2$), 3.18 (br t, *J* = 6.1 Hz, 2H, $NHCH_2$), 2.33 (t, *J* = 6.1 Hz, 2H, CH_2NMe), 2.02 [s, 6H, $N(CH_3)_2$], 1.73 (quin, *J* = 6.1 Hz, 2H, $-CH_2$); ^{13}C NMR (101 MHz, CDCl3): δ 147.6 and 146.1 (C 4 and C 6), 144.7 (C 2′), 137.3 (C 1''), 129.5 (C 3''), 129.2 (C 5′), 129.0 (C 5), 127.3 (C 4''), 126.1 (C 4′), 125.4 and 125.36 (C-1 and C 3), 123.7 (C 2''), 121.6 (C 2′), 116.3 (C 6′), 110.8 (C 3′), 96.1 (C 2), 59.2 (CH_2NMe), 45.3 [$N(CH_3)_2$], 44.0 ($NHCH_2$), 25.5 ($-CH_2-$); HRMS (ESI-TOF+): m/z calculated for $C_{23}H_{27}N_6O_5$: 451.2094; found: 451.2088 (MH+).

N^1-[2-(3-Diethylaminopropyl)aminophenyl]-N^3-phenyl-4,6-dinitro-1,3-phenylenediamine **(7c)**. Maroon crystals (2.11 g, 82%); R_f 0.36 [10% (v/v) methanol: chloroform]; mp. 181.4-183.4 °C (ethanol). ^1H NMR (400 MHz, $CDCl_3$): δ 9.66 (br s, 1H, C3-NH), 9.24 (s, 1H, H 5), 9.17 (br s, 1H, C1-NH), 7.22 – 7.15 (m, 2H, H 3''), 7.10 – 7.01 (m, 4H, H 2'', H 4', H 5′), 6.92 (br dd, *J* = 8.0, 1.0 Hz, 1H, H 4''), 6.59 – 6.53 (m, 2H, H 3′, H 6′), 6.12 (s, 1H, H 2), 5.10 (br s, 1H, $NHCH_2$), 3.00 (br t, *J* = 6.4 Hz, 2H, $NHCH_2$), 2.44 (t, *J* = 6.3 Hz, 2H, $-CH_2NEt_2$), 2.37 [q, *J* = 7.2 Hz, 4H, $N(CH_2CH_3)_2$], 1.68 (quin, *J* = 6.3 Hz, 2H, $-CH_2$), 0.83 [t, *J* = 7.0 Hz, 6H, $N(CH_2CH_3)_2$]; ^{13}C NMR (101 MHz, $CDCl_3$): δ 147.7 and 146.2 (C 4 and C 6), 144.4 (C 2′), 137.2 (C 1''), 129.4 (C 3''), 129.1 (C 5′), 129.0 (C 5), 127.2 (C 4''), 126.1 (C 4′), 125.5 and 125.4 (C-1 and C 3), 123.8 (C 2''), 121.8 (C 2′), 116.6 (C 6′), 111.2 (C 3′), 96.2 (C 2), 51.5 (CH_2NEt_2), 46.9 [$N(CH_2CH_3)_2$], 43.5 ($NHCH_2$), 25.8 ($-CH_2$), 11.0 [$N(CH_2CH_3)_2$]; HRMS (ESI-TOF+): m/z calculated for $C_{25}H_{29}N_6O_4$: 477.2250; found: 477.2242 (MH+).

N^1-[2-(2-Dimethylaminoethyl)aminophenyl]-N^3-4-methylphenyl-4,6-dinitro-1,3-phenylenediamine **(7d)**. Fluffy bright orange solid (3.25 g, 57%); R_f 0.35 [50% (v/v) methanol: ethyl acetate]; mp. 137.9-139.9 °C (chloroform/hexane). ^1H NMR (400 MHz, CDCl3): δ 9.68 (br s, 1H, C3-NH), 9.31 (s, 1H, H 5), 9.24 (br s, 1H, C1-NH), 7.15 (ddd, *J* = 8.24, 7.12, 1.83 Hz, 1H, H 5′), 7.05 (d, *J* = 8.2 Hz, 2H, H 3''), 7.01 (dd, *J* = 7.9, 1.2 Hz, 1H, H 4′), 6.97 (d, *J* = 8.2 Hz, 2H, H 2''), 6.69-6.63 (m, 2H, H 3', H 6'), 6.16 (s, 1H, H 2), 4.82 (br s, 1H, $NHCH_2$), 3.11 (q, *J* = 4.9 Hz, 2H, $NHCH_2$), 2.46 (t, *J* = 6.1 Hz, 2H, CH_2NMe_2), 2.30 (s, 3H, C-4″ CH_3), 2.15 [s, 6H, $N(CH_3)_2$]; ^{13}C NMR (101 MHz, $CDCl_3$): δ 147.6 (C 6), 146.7 (C 4), 144.1 (C 1′), 136.1 (C 4''), 134.6 (C 1''), 130.0 (C 3''), 129.1 (C 5), 128.9 (C 5′), 127.1 (C 2″), 125.4 (C 3), 125.3 (C 1), 124.0 (C 4′), 122.5 (C 2′), 117.1 (C 6′), 111.9 (C 3′), 95.9 (C 2), 57.8 (CH_2N), 45.2 [$N(CH_3)_2$], 41.1 ($NHCH_2$), 20.9 (C-4′ CH_3); HRMS (ESI-TOF+): m/z calculated for $C_{23}H_{27}N_6O_4$: 451.2094; found: 451.2108 (MH+).

N^1-[2-(3-Dimethylaminopropyl)aminophenyl]-N^3-4-methylphenyl-4,6-dinitro-1,3-phenylenediamine **(7e)**. Maroon crystals (2.54 g, 80%); R_f 0.37 [50% (v/v) methanol: ethyl acetate]; mp. 148.7-150.7 °C (ethanol). ^1H NMR (400 MHz, $CDCl_3$): δ 9.70 (br s, 1H, C3-NH), 9.32 (s, 1H, H 5), 9.25 (br s, 1H, C1-NH), 7.13 (td, *J* = 7.8, 1.5 Hz, 1H, H 5'), 7.05 (d, *J* = 8.2 Hz, 2H, H 3''), 7.00 (d, *J* = 9.1 Hz, 2H, H 2''), 7.02 – 6.96 (m, 1H, H 4′), 6.60 (d, *J* = 7.6 Hz, 1H, H-3′), 6.60 (dd, *J* = 6.4, 1.2 Hz, 1H, H 6′), 6.13 (s, 1H, H 2), 5.60 (br s, 1H, $NHCH_2$), 3.17 (t, *J* = 6.3Hz, 2H, $NHCH_2$), 2.34 (t, *J* = 6.1 Hz, 2H, CH_2NMe), 2.30 (s, 3H, Ph- CH_3), 2.03 [s, 6H, $N(CH_3)_2$], 1.73 (quin, *J* = 6.1 Hz, 2H, $-CH_2$); ^{13}C NMR (101 MHz, $CDCl_3$): δ 147.5 (C 4), 146.5 (C 6), 144.6 (C 1'), 136.0 (C 4''), 134.6 (C 1''), 130.0 (C 3''), 129.0 (C 5, C 5'), 127.2 (C 2''), 125.3 (C 1), 125.25 (C 3), 123.7 (C 4''), 121.7 (C 1'), 116.4 (C 6'), 111.0 (C 3'), 95.9 (C 2), 59.2 (CH_2NMe_2), 45.2 [$N(CH_3)_2$], 44.0 ($NHCH_2$), 25.6 ($-CH_2$), 20.9 (C-4″ CH_3); HRMS (ESI-TOF+): m/z calculated for $C_{24}H_{29}N_6O_5$: 465.2250; found: 465.2263 (MH+).

N^1-[2-(3-Diethylaminopropyl)aminophenyl]-N^3-4-methylphenyl-4,6-dinitro-1,3-phenylenediamine **(7f)**. Maroon crystals (1.09 g, 81%); R_f 0.24 [10% (v/v) methanol: chloroform]; mp. 101.7-103.7 °C (ethanol). ^1H NMR (400 MHz, $CDCl_3$): δ 9.69 (br s, 1H, C3-NH), 9.31 (s, 1H, H 5), 9.23 (br s, 1H, C1-NH), 7.12 (td, *J* = 7.8, 1.4 Hz, 1H, H 5′), 7.04 (d, *J* = 8.9 Hz, 2H, H 3''), 6.99 (d, *J* = 8.5 Hz, 3H, H 2'', H 4′), 6.64 (d, *J* = 7.9 Hz, 1H, H 6′), 6.61 (m, 1H, H 3′), 6.14 (s, 1H, H 2), 5.08 (br s, 1H, $NHCH_2$), 3.17 (t, *J* = 6.3 Hz, 2H, $NHCH_2$), 2.48 (t, *J* = 6.3 Hz, 2H, CH_2NMe), 2.41 [q, *J* = 7.0 Hz, 4H, $N(CH_2CH_3)_2$], 2.30 (s, 3H, C-4″ CH_3), 1.72 (quin, *J* = 6.3 Hz, 2H, $-CH_2$), 0.87 [t, *J* = 7.0 Hz, 6H, $N(CH_2CH_3)_2$]; ^{13}C NMR (101 MHz, $CDCl_3$): δ 147.6 (C 6), 146.6 (C 4), 144.5 (C 1') 136.1 (C 4''), 134.6 (C 1''), 130.0 (C 3''), 129.0 (C 5), 128.96 (C 5'), 127.2 (C 2''), 125.37 (C 1), 125.35 (C 3), 123.8 (C 4'), 121.9 (C 2'), 116.5 (C 6'), 111.2 (C 3'), 96.1 (C 2), 51.7 (CH_2NMe_2), 47.0

[N(CH_2CH_3)$_2$], 43.7 ($NHCH_2$), 26.0 (-CH_2), 20.9 (C-4″ CH_3), 11.2 [N(CH_2CH_3)$_2$]; HRMS (ESI-TOF+): m/z calculated for $C_{26}H_{33}N_6O_4$: 493.2563; found: 493.2546 (MH+).

N^1-[2-(2-Dimethylaminoethyl)aminophenyl]-N^3-4-ethoxyphenyl-4,6-dinitro-1,3-phenylenediamine **(7g)**. Bright orange wax (3.49 g, 50%); *R*$_f$ 0.26 [10% (v/v) methanol:chloroform]; mp. 133.3-135.3 °C (chloroform/hexane). ^{1}H NMR (400 MHz, $CDCl_3$): δ 9.60 (br s, 1H, C3-NH), 9.31 (s, 1H, H 5), 9.23 (br s, 1H, C1-NH), 7.14 (td, *J* = 7.9, 1.5 Hz, 1H, H 5′), 6.99-6.95 (m, 1H, H 3′), 6.98 (d, *J* = 8.5 Hz, 2H, H 2″), 6.77 (d, *J* = 9.2 Hz, 2H, H 3''), 6.65-6.59 (m, 2H, H 4′, H 6′), 6.04 (s, 1H, H 2), 4.42 (br t, *J* = 5.0 Hz, 1H, $NHCH_2$), 4.00 (q, *J* = 6.8 Hz, 2H, OCH_2CH_3), 3.10 (dt, *J* = 9.2, 5.8 Hz, 2H, $NHCH_2$), 2.46 (t, *J* = 6.3 Hz, 2H, -CH_2NMe_2), 2.15 [s, 6H, N(CH_3)$_2$], 1.41 (t, *J* = 6.9 Hz, 3H, OCH_2CH_3); ^{13}C NMR (101 MHz, $CDCl_3$): δ 157.3 (C 4''), 147.6 (C 4), 147.3 (C 6), 144.1 (C 1′), 129.7 (C 1''), 129.1 (C 5), 128.9 (C 5′), 127.1 (C 3′), 126.0 (C 2''), 125.3 (C 3), 125.2 (C 1), 122.5 (C 2′), 117.0 (C 6′), 115.3 (C 3''), 111.9 (C 4'), 95.7 (C 2), 63.8 (OCH_2CH_3), 57.8 (CH_2NMe_2), 45.2 [N(CH_3)$_2$], 41.1 ($NHCH_2$), 14.7 (OCH_2CH_3); HRMS (ESI-TOF+): m/z calculated for $C_{24}H_{29}N_6O_5$: 481.2199; found: 481.2205 (MH+).

N^1-[2-(3-Dimethylaminopropyl)aminophenyl]-N^3-4-ethoxyphenyl-4,6-dinitro-1,3-phenylenediamine **(7h)**. Maroon crystals (1.94 g, 84%); *R*$_f$ 0.38 [50% (v/v) methanol: ethyl acetate]; mp. 108.5-110.5 °C (ethanol). ^{1}H NMR (400 MHz, $CDCl_3$): δ 9.61 (br s, 1H, C3-NH), 9.32 (s, 1H, H 5), 9.24 (br s, 1H, C1-NH), 7.14 (td, *J* = 8.5, 2.3 Hz, 1H, H 5′), 7.02 (d, *J* = 8.6 Hz, 2H, H 2″), 7.00-6.96 (m, 1H, H 3'), 6.78 (d, *J* = 9.0 Hz, 2H, H 3''), 6.65-6.60 (m, 2H, H 4′, H 6′), 6.02 (s, 1H, H 2), 5.33 (br s, 1H, $NHCH_2$), 4.01 (q, *J* = 7.0 Hz, 2H, OCH_2CH_3), 3.19 (t, *J* = 6.3 Hz, 2H, $NHCH_2$), 2.44 (t, *J* = 6.1 Hz, 2H, -CH_2NMe_2), 2.14 [s, 6H, N(CH_3)$_2$], 1.79 (quin, *J* = 6.2 Hz, 2H, -CH_2-), 1.42 (t, *J* = 7.0 Hz , 3H, OCH_2CH_3); ^{13}C NMR (101 MHz, $CDCl_3$): δ 157.2 (C 4''), 147.4 (C 4), 147.1 (C 6), 144.6 (C 1′), 129.7 (C 1''), 129.01 (C 5), 128.98 (C 5′), 127.2 (C 3′), 125.8 (C 2''), 125.2 (C 3), 125.1 (C 1), 121.7 (C 2′), 116.3 (C 6′), 115.3 (C 3''), 110.9 (C 4'), 95.7 (C 2), 63.8 (OCH_2CH_3), 59.1 (CH_2NMe_2), 45.3 [N(CH_3)$_2$], 43.8 ($NHCH_2$), 25.6 (-CH_2), 14.7 (OCH_2CH_3); HRMS (ESI-TOF+): m/z calculated for $C_{25}H_{31}N_6O_5$: 495.2356; found: 495.2363 (MH+).

N^1-[2-(3-Diethylaminopropyl)aminophenyl]-N^3-4-ethoxyphenyl-4,6-dinitro-1,3-phenylenediamine **(7i).** Dark orange solid (1.15 g, 69%); *R*$_f$ 0.32 [10% (v/v) methanol:chloroform]; mp. 90.9-92.9 °C (ethanol). ^{1}H NMR (400 MHz, $CDCl_3$): δ 9.60 (br s, 1H, C3-NH), 9.30 (s, 1H, H 5), 9.21 (br s, 1H, C1-NH), 7.13 (td, *J* = 7.9, 1.5 Hz, 1H, H 5′), 7.01 (d, *J* = 8.5 Hz, 2H, H 3''), 6.97 (dd, *J* = 8.1, 1.4 Hz, 1H, H 3′), 6.77 (d, *J* = 8.9 Hz, 2H, H 2''), 6.64-6.59 (m, 2H, H 4′, H 6′), 6.02 (s, 1H, H 2) 5.00 (br s, 1H, $NHCH_2$), 4.00 (q, *J* = 6.9 Hz, 2H, OCH_2CH_3), 3.15 (t, *J* = 6.4 Hz, 2H, $NHCH_2$), 2.48 (t, *J* = 6.3 Hz, 2H, -CH_2NEt_2), 2.40 [q, *J* = 6.3 Hz, 4H, N(CH_2CH_3)$_2$], 1.72 (quin, *J* = 6.3 Hz, 2H, -CH_2-), 1.42 (t, *J* = 7.0 Hz, 3H, OCH_2CH_3), 0.87 [t, *J* = 7.2 Hz, 6H, N(CH_2CH_3)$_2$]; ^{13}C NMR (101 MHz, CDCl3): δ 157.2 (C 4''), 147.5 (C 4), 147.2 (C 6), 144.4 (C 1′), 129.7 (C 1''), 129.1 (C 5), 128.9 (C 5′), 127.2 (C 3′), 125.9 (C 1), 125.3 (C 3), 125.2 (C 2''), 121.9 (C 2′), 116.5 (C 6′), 115.5 (C 3''), 111.2 (C 4′), 95.8 (C 2), 63.8 (OCH_2CH_3), 51.6 (CH_2NEt_2), 46.9 [N(CH_2CH_3)$_2$], 43.6 ($NHCH_2$), 26.0 (-CH_2), 14.7 (OCH_2CH_3), 11.2 [N(CH_2CH_3)$_2$]; HRMS (ESI-TOF+): m/z calculated for $C_{27}H_{33}N_6O_5$: 521.2512; found: 521.2511 (MH+).

N^1-[2-(2-Dimethylaminoethyl)amino-5-methylphenyl]-N^3-4-methylphenyl-4,6-dinitro-1,3-phenylenediamine **(7j)**. Bright orange crystals (1.54 g, 67%); *R*$_f$ 0.41 [5% (v/v) methanol: chloroform]; mp. 126-128 °C (ethanol). ^{1}H NMR (400 MHz, $CDCl_3$): δ 9.68 (br s, 1H, C3-NH), 9.32 (s, 1H, H 5), 9.29 (br s, 1H, C1-NH), 7.08 (d, *J* = 7.9 Hz, 2H, H 3''), 7.00 (d, *J* = 8.5 Hz, 2H, H 2''), 6.98 (dd, *J* = 8.2, 2.1 Hz, 1H, H 5′), 6.85 (d, *J* = 1.8 Hz, 1H, H 3′), 6.59 (d, *J* = 8.5 Hz, 1H, H 6′), 6.23 (s, 1H, H 2), 4.22 (br s, 1H, $NHCH_2$), 3.09 (td, *J* = 6.1, 4.6 Hz, 2H, $NHCH_2$), 2.46 (t, *J* = 6.1 Hz, 2H, -CH_2NMe_2), 2.31 (s, 3H, C4″-CH_3), 2.21 (s, 3H, C4′-CH_3), 2.15 [s, 6H, N(CH_3)$_2$]; ^{13}C NMR (101 MHz, $CDCl_3$): δ 147.4 (C 4), 146.8 (C 6), 141.5 (C 1'), 136.2 (C 1''), 134.7 (C 4''), 130.0 (C 3''), 129.1 (C 5), 129.1 (C 5'), 127.0 (C 3'), 126.6 (C 4'), 125.4 (C 1), 125.3 (C 3), 124.2 (C 2''), 122.7 (C 2'), 112.4 (C 6'), 95.9 (C 2), 57.9 (CH_2NMe_2), 45.2 [N(CH_3)$_2$)], 41.5 ($NHCH_2$), 20.9 (C4''-CH_3), 20.2 (C5'-CH_3); HRMS (ESI-TOF+): m/z calculated for $C_{24}H_{29}N_6O_4$: 465.2250; found: 465.2224 (MH+).

N^1-[2-(3-Dimethylaminopropyl)amino-5-methylphenyl]-N^3-4-methylphenyl-4,6-dinitro-1,3-phenylenediamine **(7k)**. Dark maroon crystals (3.61 g, 73%); R_f 0.32 [50% (v/v) methanol:ethyl acetate]; mp. 217.5-219.5 °C (ethanol). ^{1}H NMR (400 MHz, $CDCl_3$): δ 9.69 (br s, 1H, C3-NH), 9.31 (s, 1H, H 5), 9.27 (br s, 1H, C1-NH), 7.08 (d, *J* = 8.2 Hz, 2H, H 3''), 7.04 (d, *J* = 8.9 Hz, 2H, H 2''), 6.95 (dd, *J* = 8.2, 2.1 Hz, 1H, H 5′), 6.82 (d, *J* = 2.1 Hz, 1H, H 6′), 6.56 (d, *J* = 8.5 Hz, 1H, H 3′), 6.19 (s, 1H, H 2), 4.51 (br s, 1H, $NHCH_2$), 3.16 (t, *J* = 6.1 Hz, 2H, $NHCH_2$), 2.35 (t, *J* = 6.1 Hz, 2H, -CH_2NMe_2), 2.31 (s, 3H, C4''-CH_3), 2.20 (s, 3H, C4'-CH_3), 2.05 [s, 6H, $N(CH_3)_2$], 1.73 (quin, *J* = 6.2 Hz, 1H, -CH_2-); ^{13}C NMR (101 MHz, $CDCl_3$): 147.3 (C 4), 146.5 (C 6), 142.1 (C 1′), 136.0 (C 1''), 134.7 (C 4''), 130.0 (C 3''), 129.3 (C 5'), 129.0 (C 5), 127.2 (C 3'), 125.9 (C 4'), 125.3 (C-3), 125.2 (C-1), 123.8 (C 2''), 121.8 (C 2'), 111.3 (C 6') 96.0 (C 2), 59.0 (CH_2NMe_2), 45.3 [$N(CH_3)_2$], 44.0 ($NHCH_2$), 25.8 (-CH_2-), 20.9 (C4''-CH_3), 20.2 (C5'-CH_3); HRMS (ESI-TOF+): m/z calculated for $C_{25}H_{31}N_6O_4$: 479.2407; found: 479.2400 (MH+).

NMR SPECTRA OF TARGETED FINAL COMPOUNDS

- ^{1}H NMR of Compound **10a**

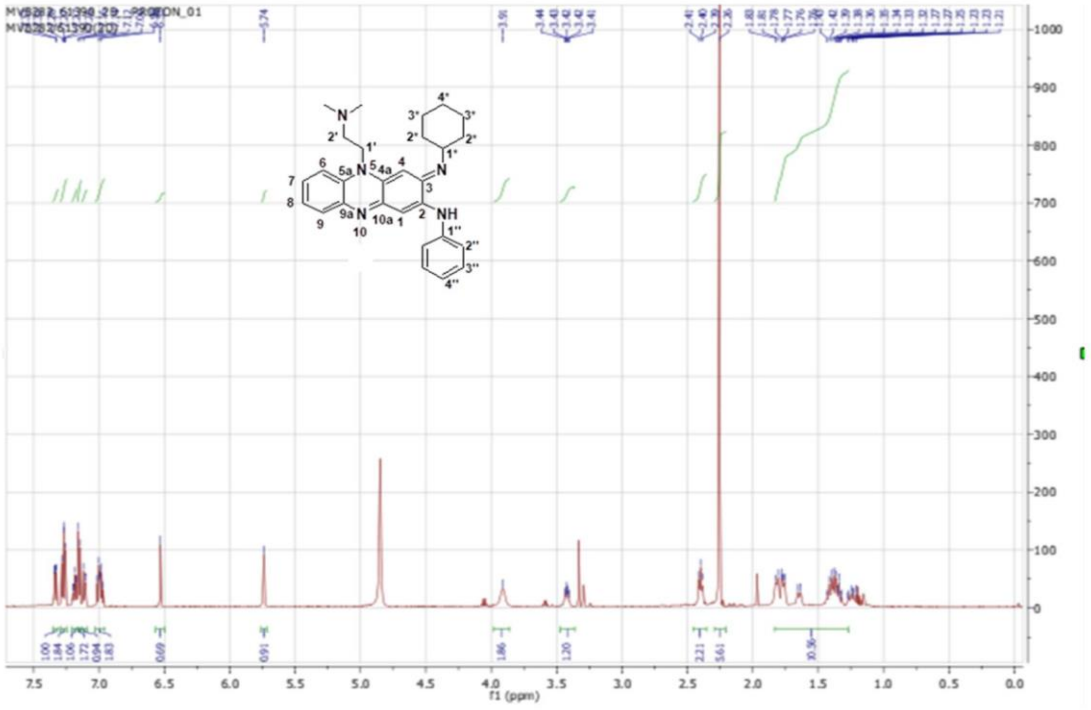

Figure S1. ^{1}H NMR spectrum of compound **10a**.

- ^{1}H NMR of Compound **10b**

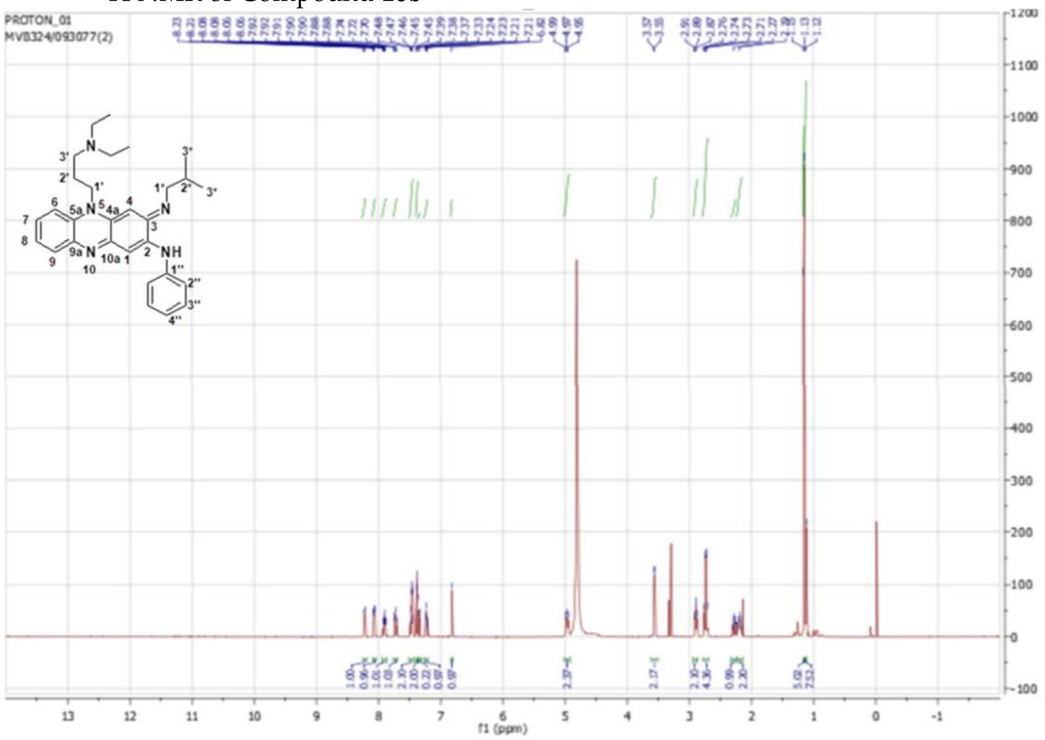

Figure S2. ^{1}H NMR spectrum of compound **10b**

^{13}C NMR of Compound **10b**

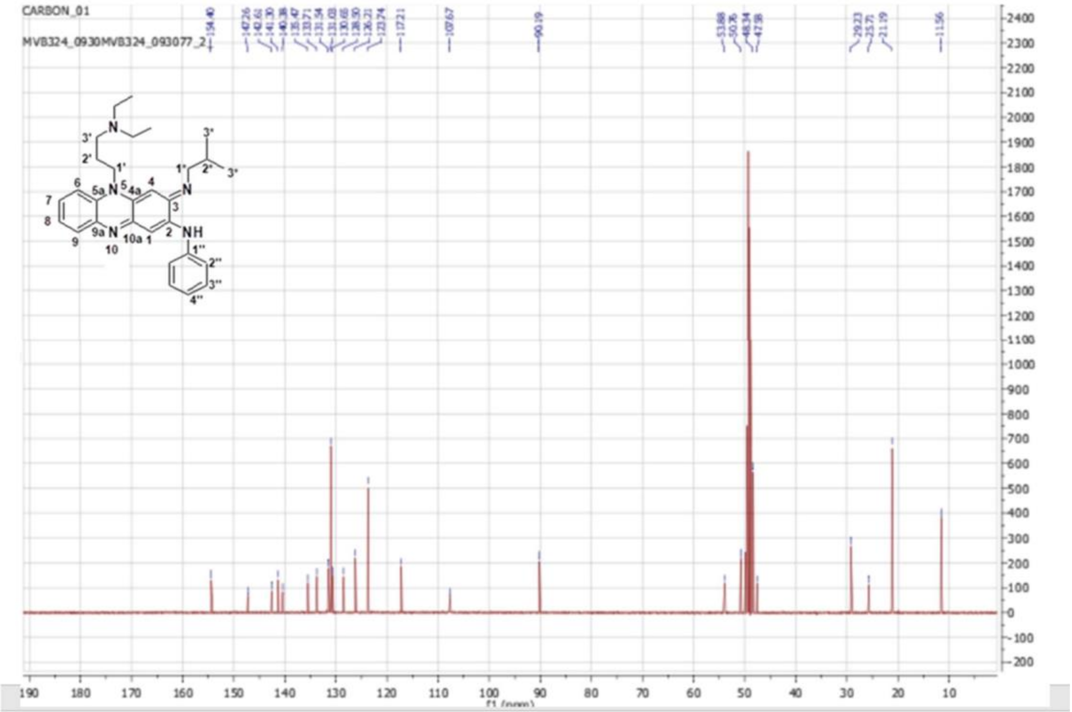

Figure S3. ^{13}C NMR spectrum of compound **10b**

- ^{1}H NMR of Compound **10c**

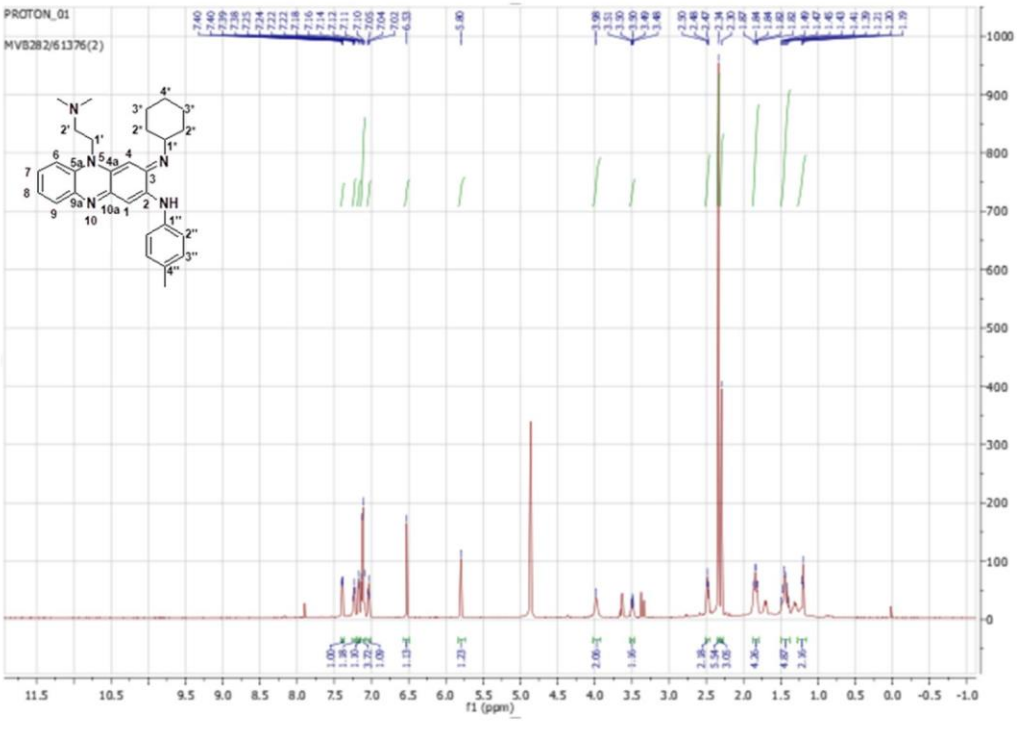

Figure S4. ^{1}H NMR spectrum of compound **10c**

- ^{13}C NMR of Compound **10c**

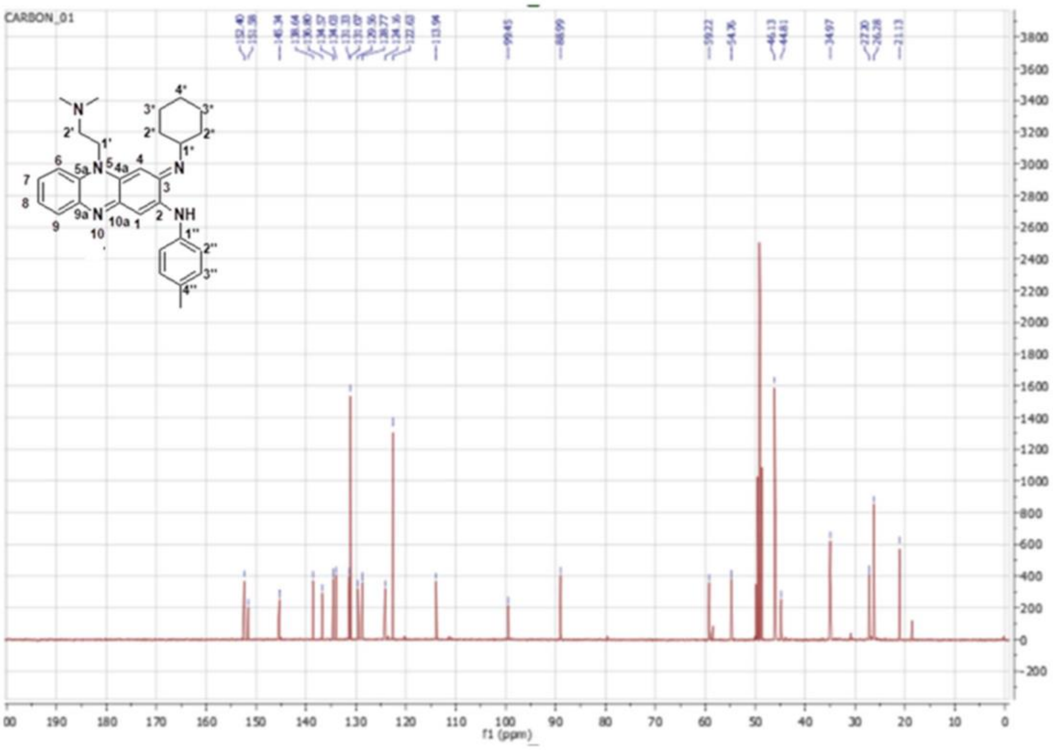

Figure S5. ^{13}C NMR spectrum of compound **10c**

- ^{1}H NMR of Compound **10d**

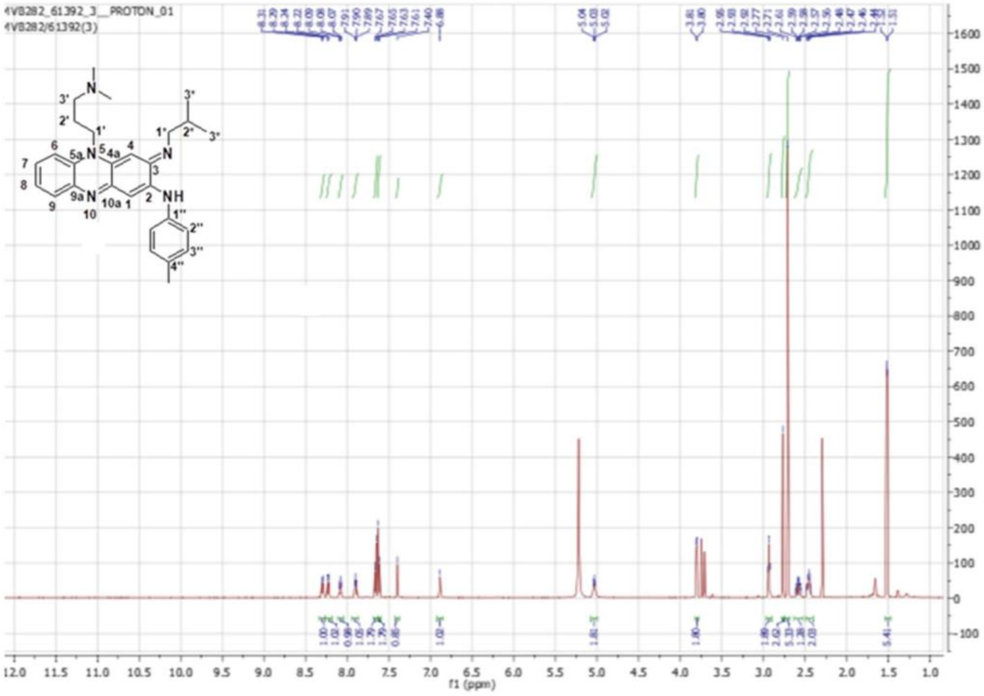

Figure S6. ^{1}H NMR spectrum of compound **10d**

- ^{13}C NMR of Compound **10d**

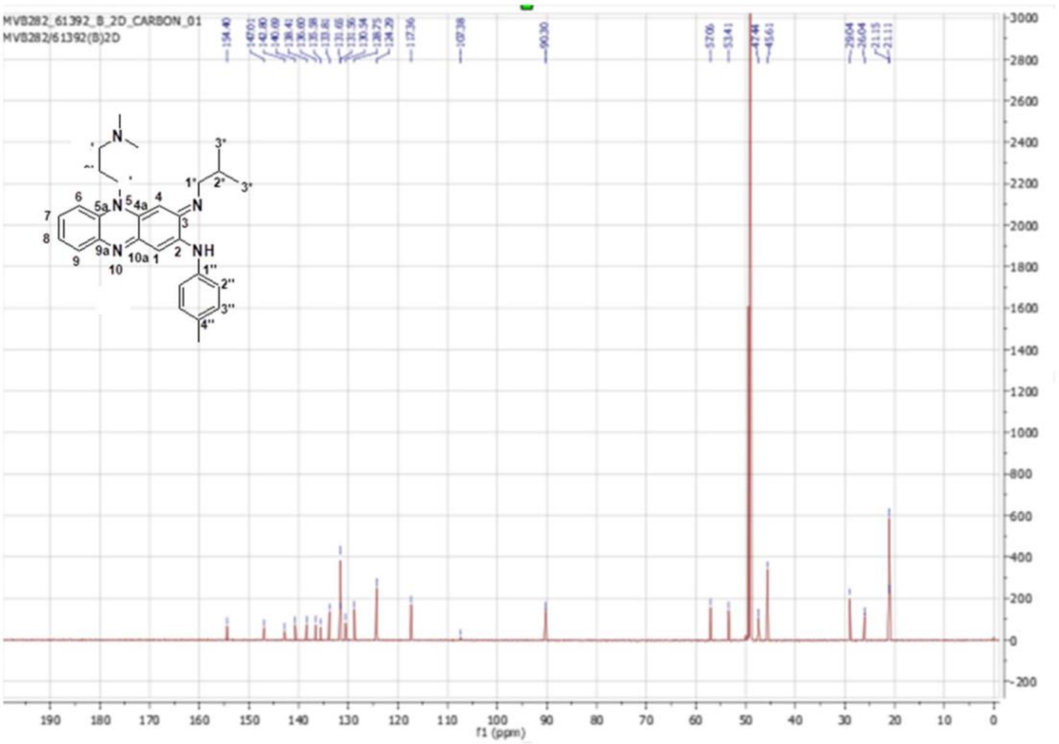

Figure S7. ^{13}C NMR spectrum of compound **10d**

- ^{1}H NMR of Compound **10e**

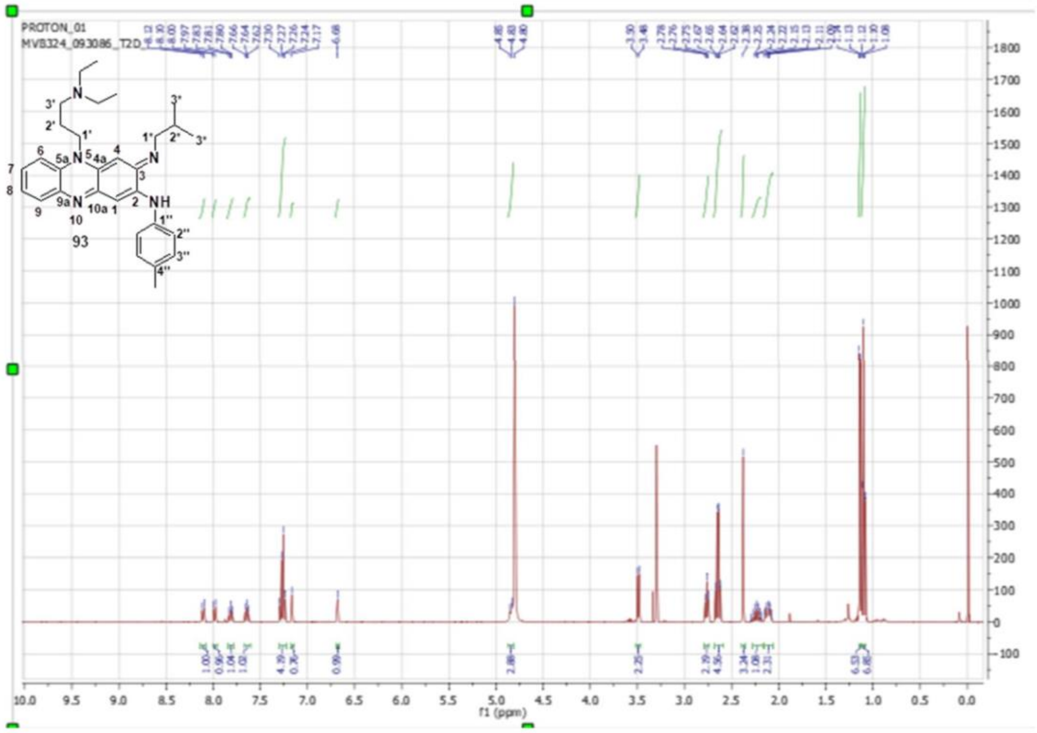

Figure S8. ^{1}H NMR spectrum of compound **10e**

- ^{13}C NMR of Compound **10e**

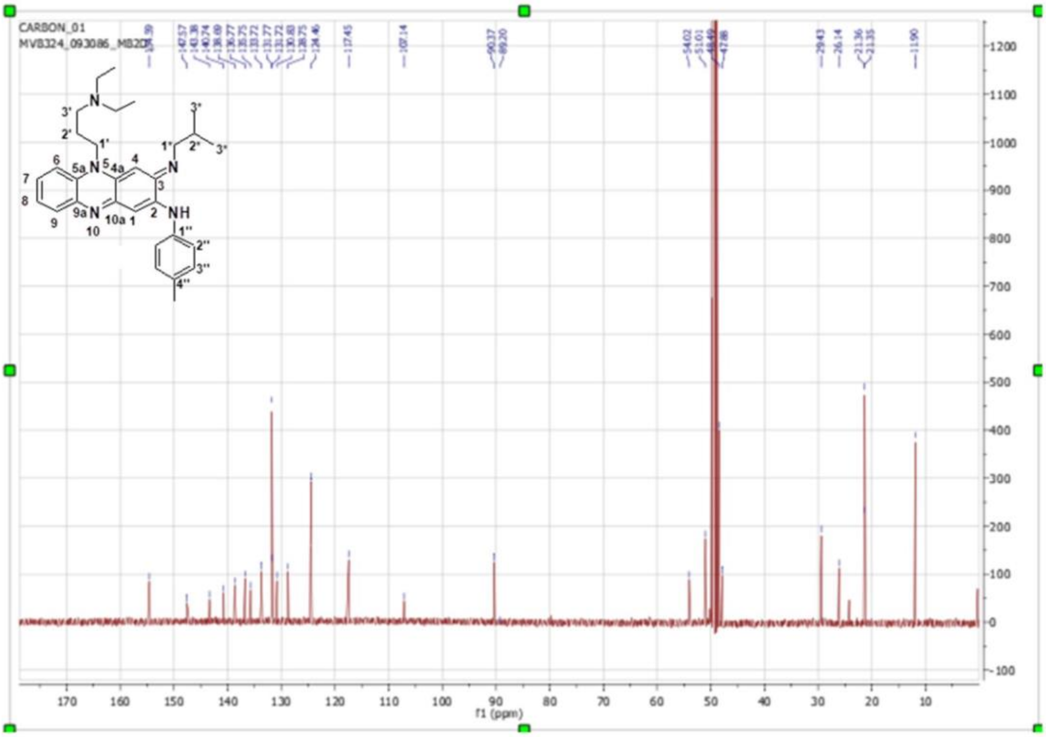

Figure S9. ^{13}C NMR spectrum of compound **10e**

- ^{1}H NMR of Compound **10f**

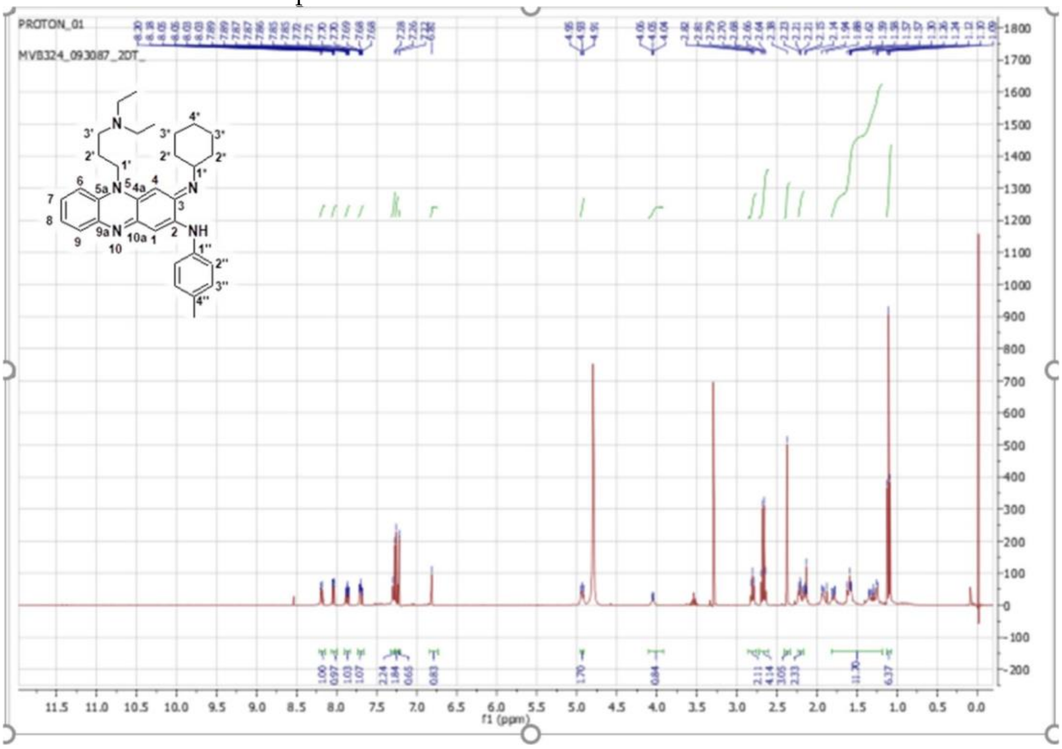

Figure S10. 1H NMR spectrum of compound **10f**

- ^{13}C NMR of Compound **10f**

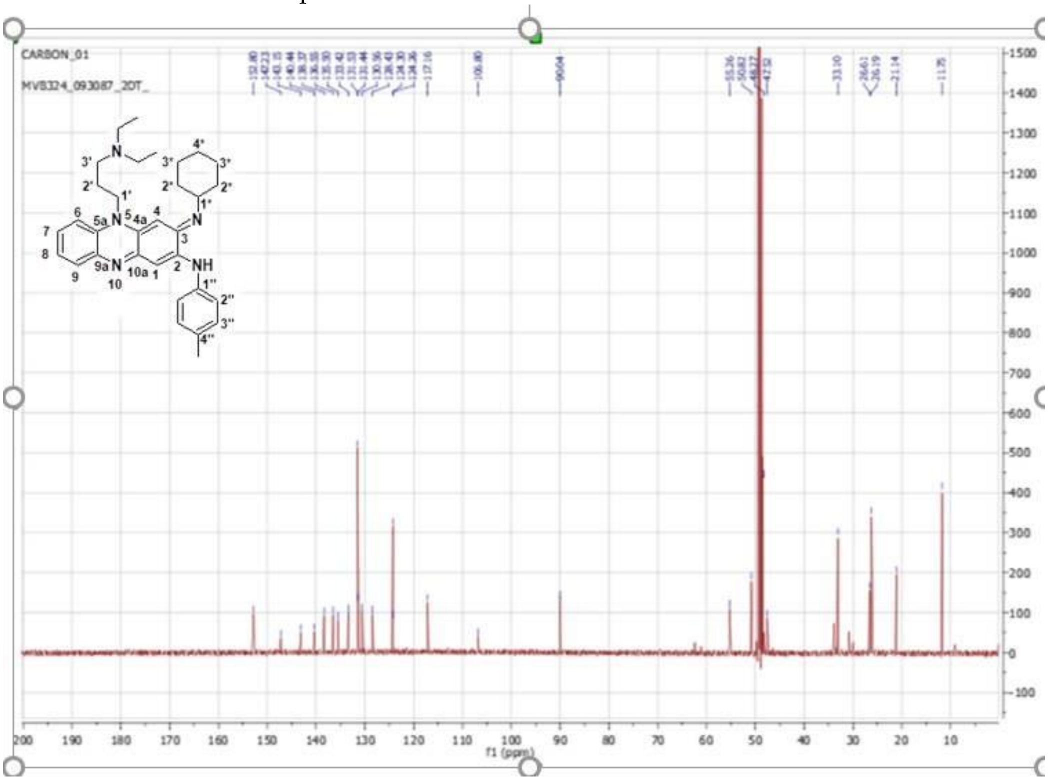

Figure S11. ^{13}C NMR spectrum of compound **10f**

• 1H NMR of Compound **10g**

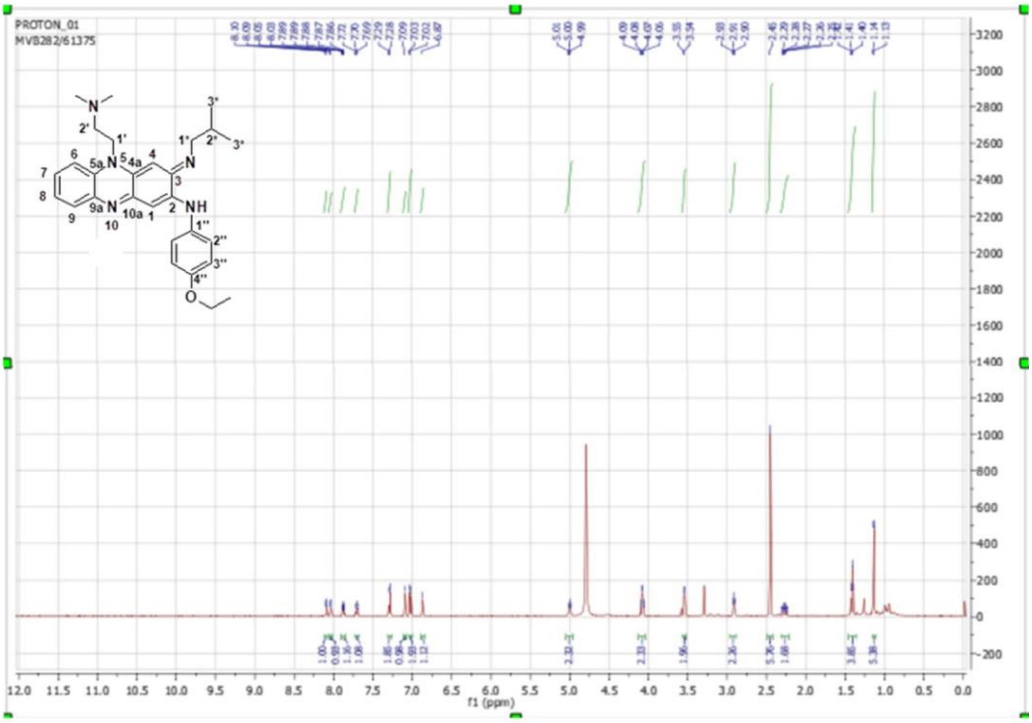

Figure S12. ^{1}H NMR spectrum of compound **10g**

• ^{13}C NMR of Compound **10g**

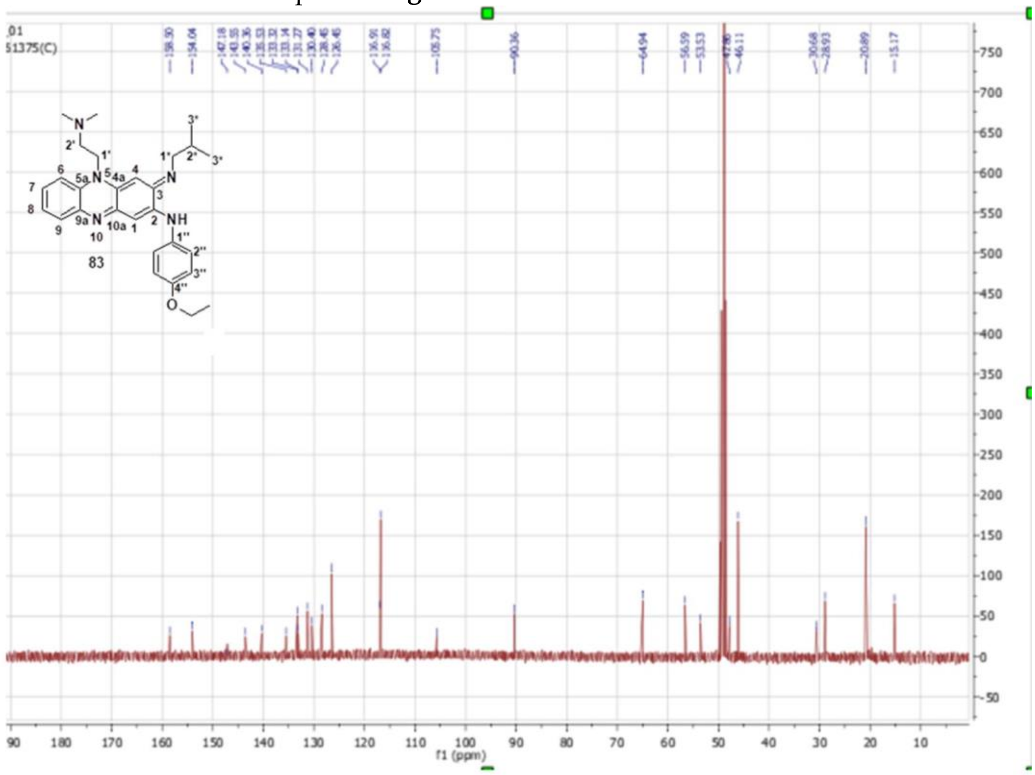

Figure S13. ^{13}C NMR spectrum of compound **10g**

- ^{1}H NMR of Compound **10h**

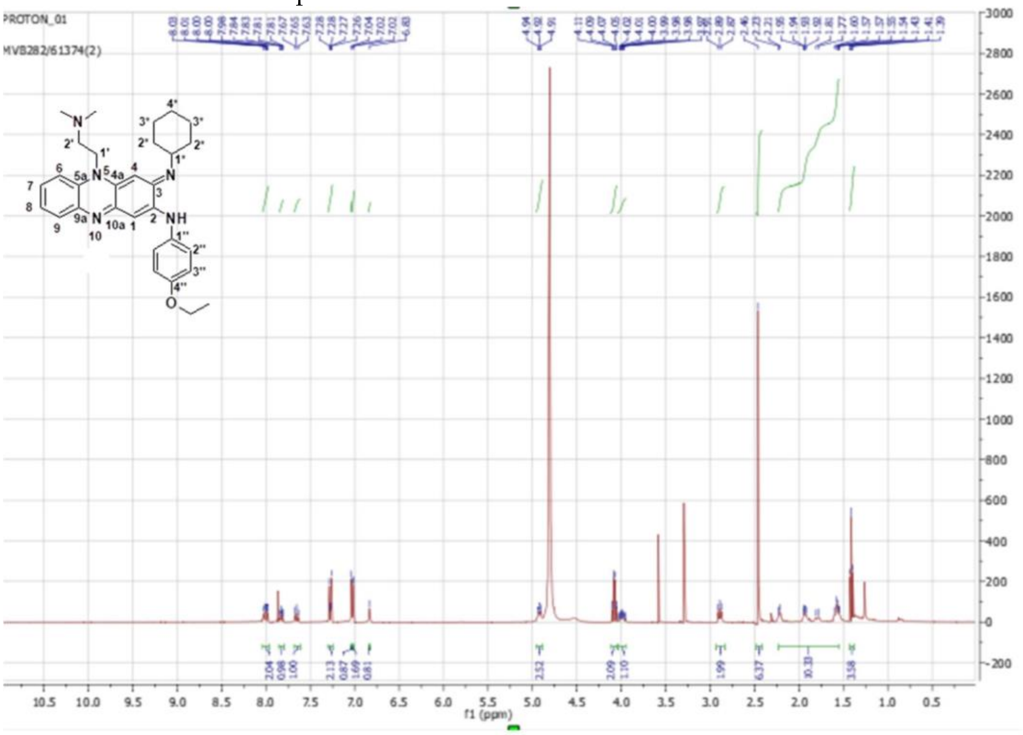

Figure S14. ^{1}H NMR spectrum of compound **10h**

- ^{13}C NMR of Compound **10h**

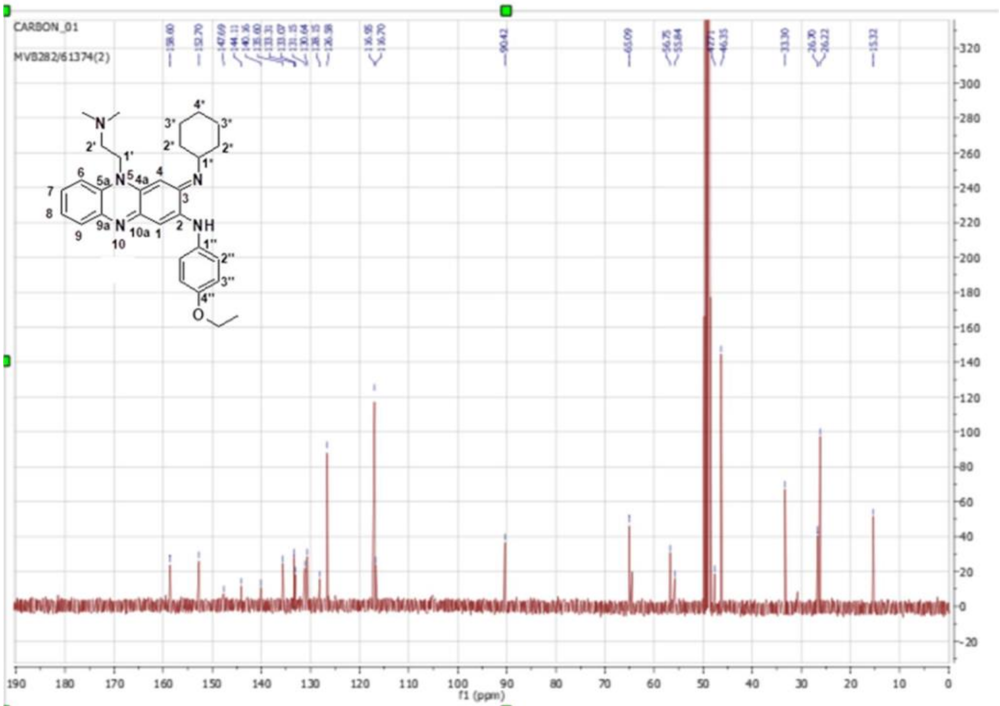

Figure S15. ^{13}C NMR spectrum of compound **10h**

- ^{1}H NMR of Compound **10i**

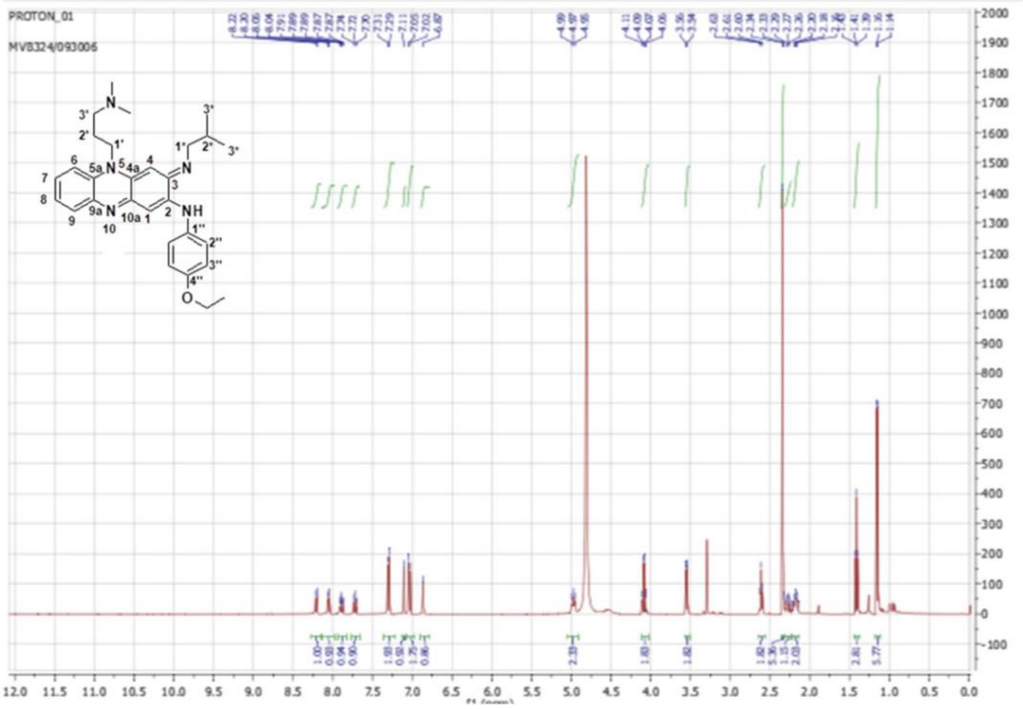

Figure S16. ^{1}H NMR spectrum of compound **10i**

- ^{13}C NMR of Compound **10i**

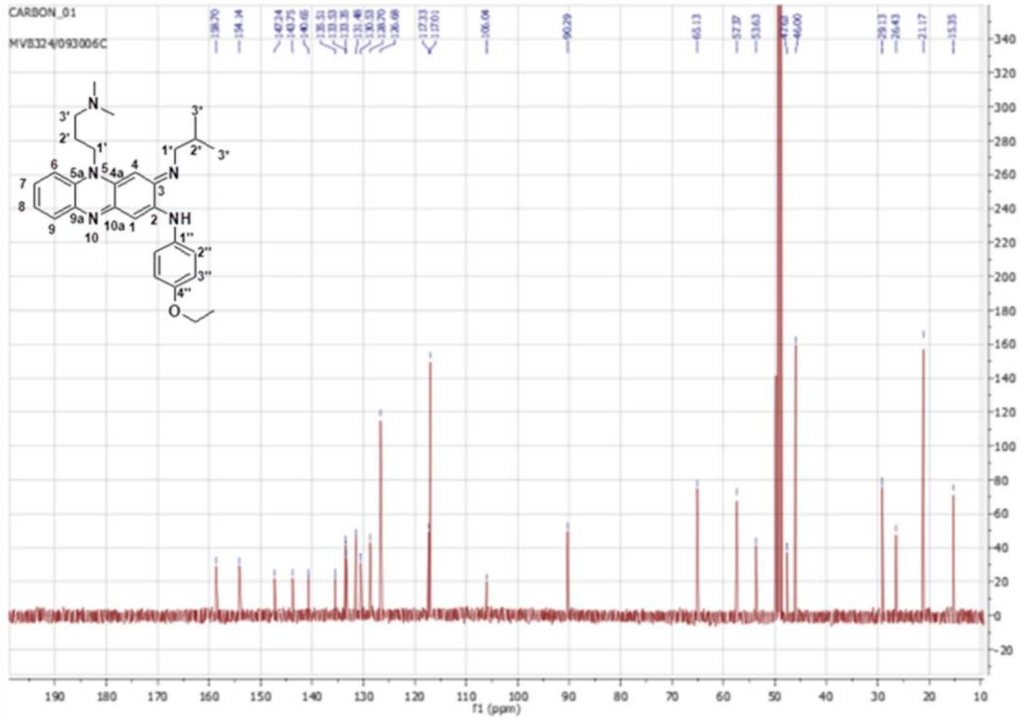

Figure S17. ^{13}C NMR spectrum of compound **10i**

- ^{1}H NMR of Compound **10J**

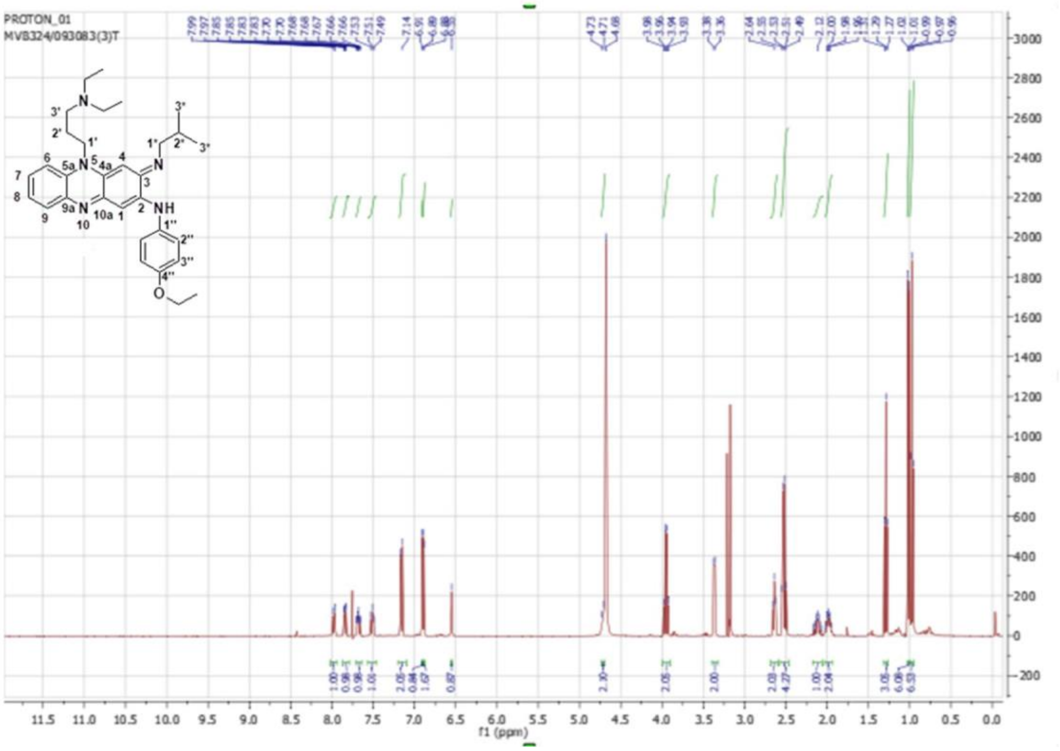

Figure S18. ^{1}H NMR spectrum of compound **10J**

- ^{1}H NMR of Compound **10K**

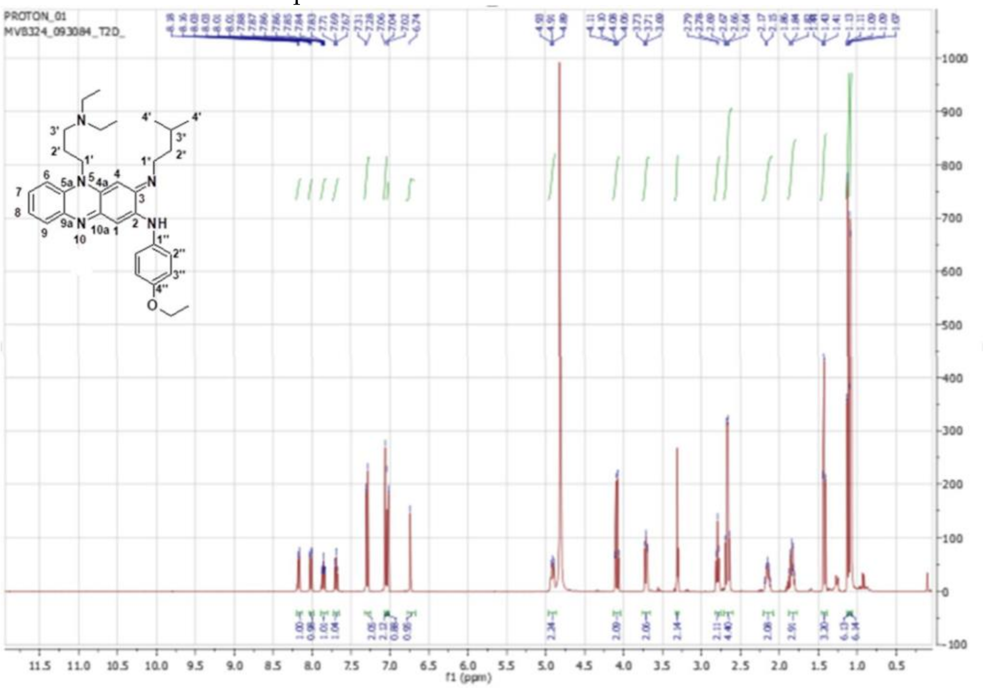

Figure S19. ^{1}H NMR spectrum of compound **10K**

- ^{13}C NMR of Compound **10K**

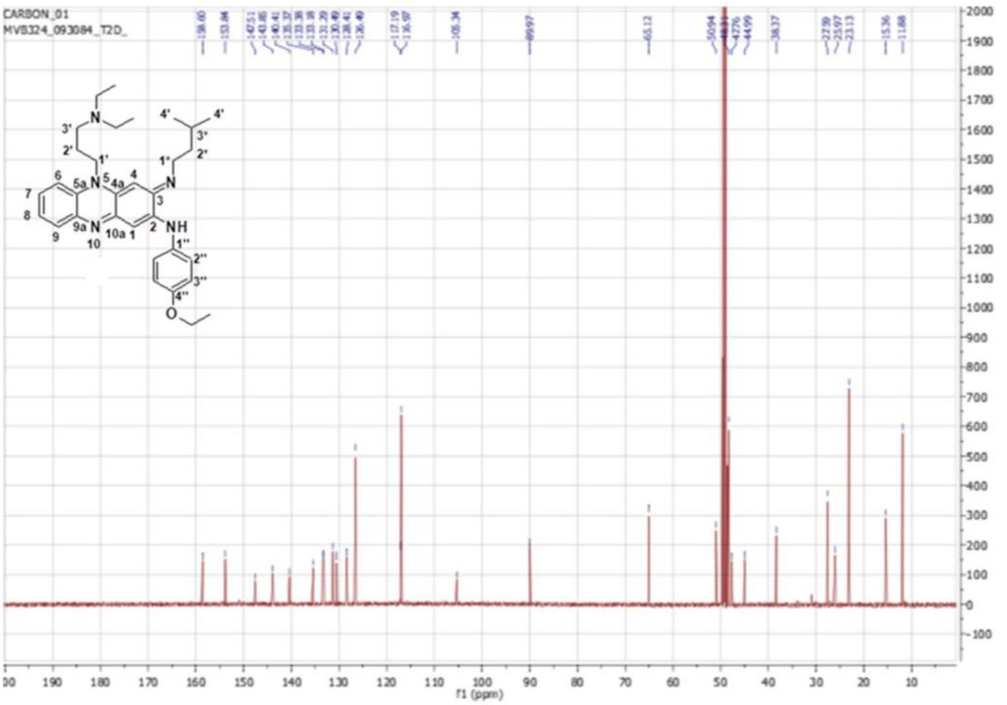

Figure S20. ^{13}C NMR spectrum of compound 10K

- ^{1}H NMR of Compound **10l**

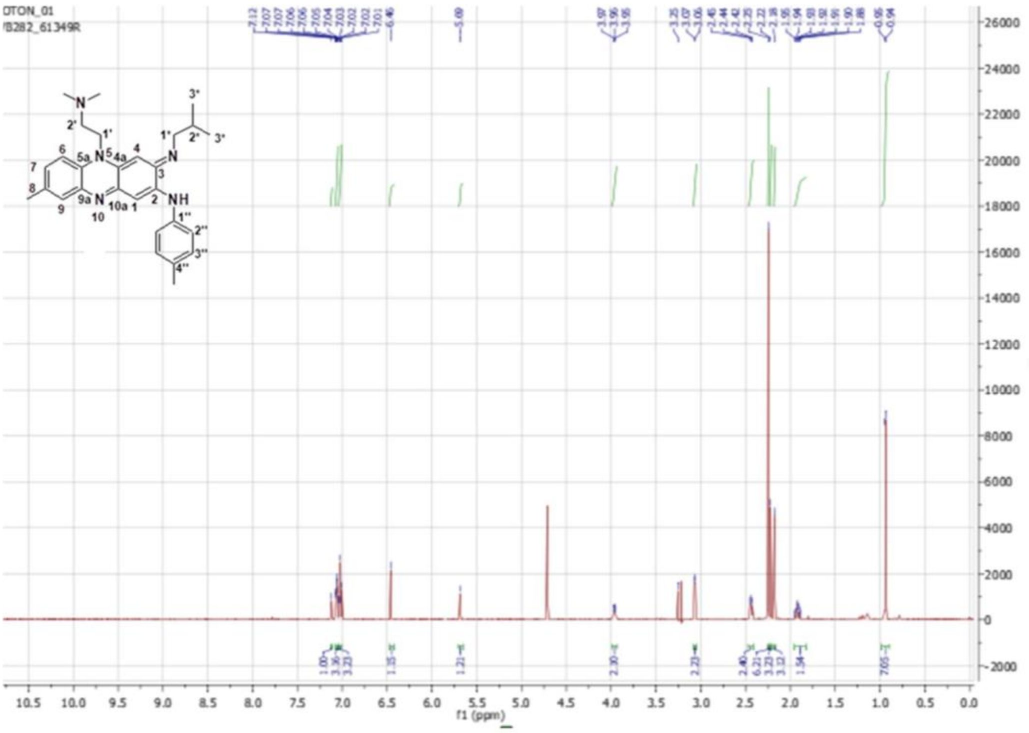

Figure S21. ^{13}C NMR spectrum of compound **10l**

- ^{13}C NMR of Compound **10l**

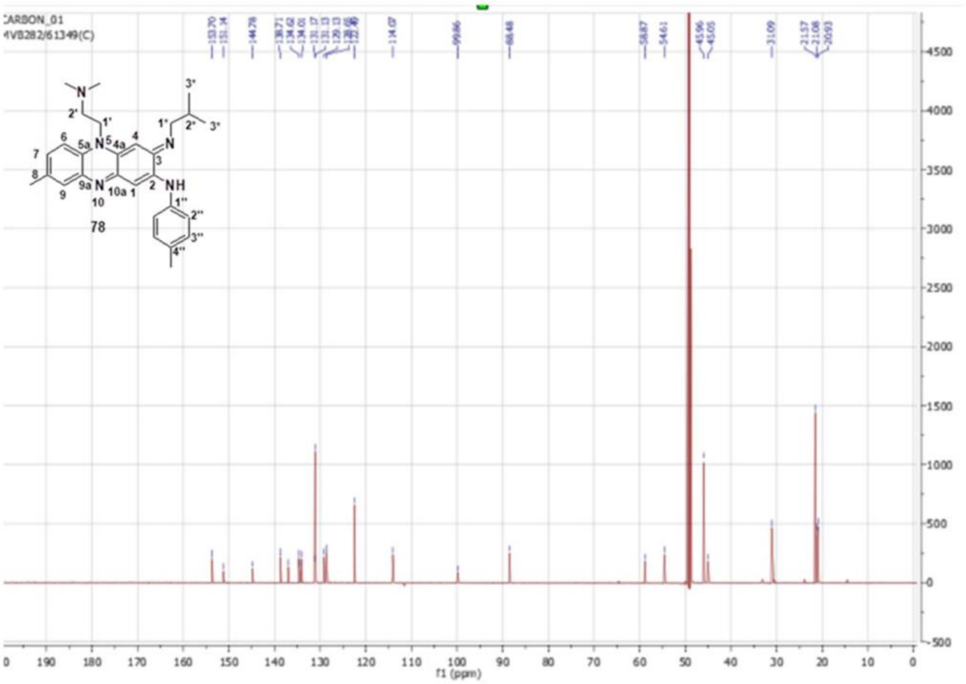

Figure S22. ^{13}C NMR spectrum of compound **10l**

- 1H NMR of Compound **10m**

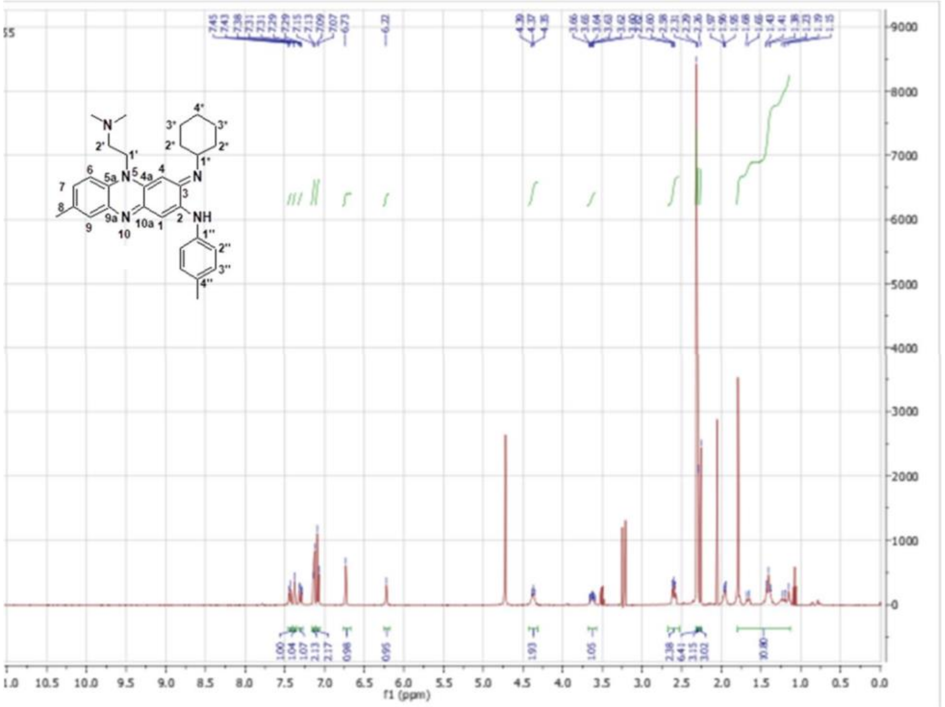

Figure S23. ^{1}H NMR spectrum of compound **10m**

- ^{13}C NMR of Compound **10m**

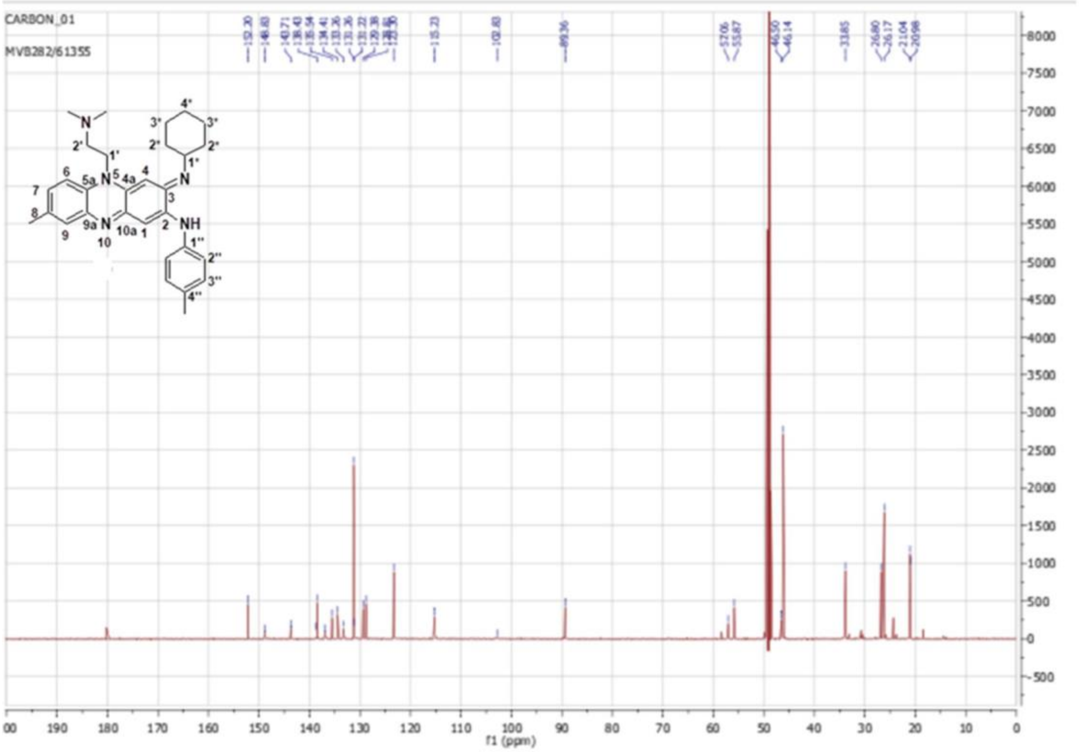

Figure S24. ^{13}C NMR spectrum of compound **10m.**

- ^{1}H NMR of Compound **10n**

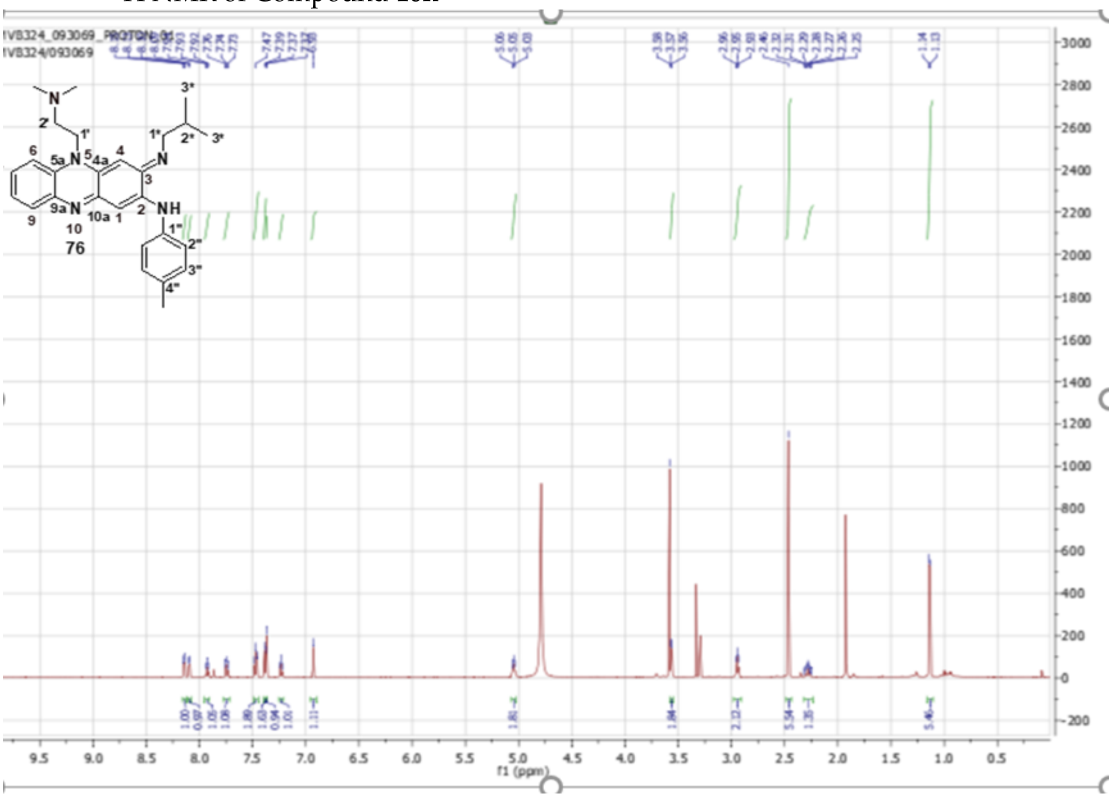

Figure S25: 1H NMR spectrum of compound **10n**

- ^{1}H NMR of compound **10p**

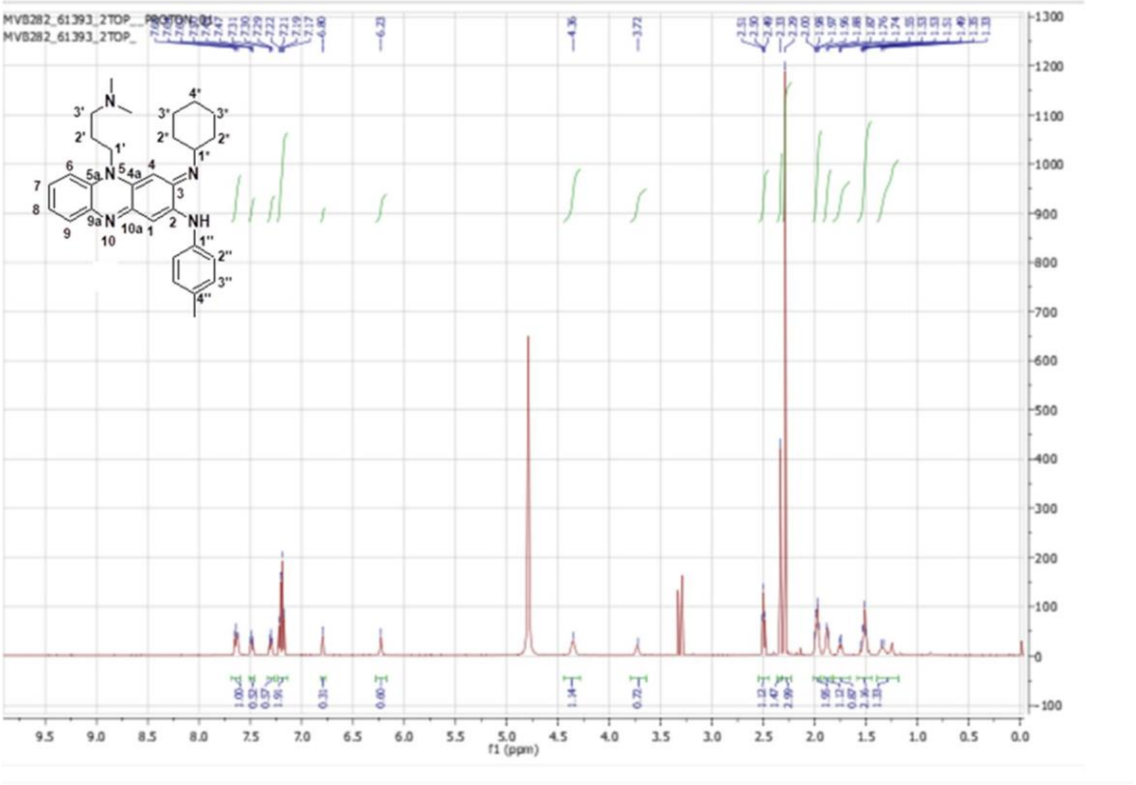

Figure S27: 1H NMR of spectrum of 10p

- **^{13}C NMR for compound 10p**

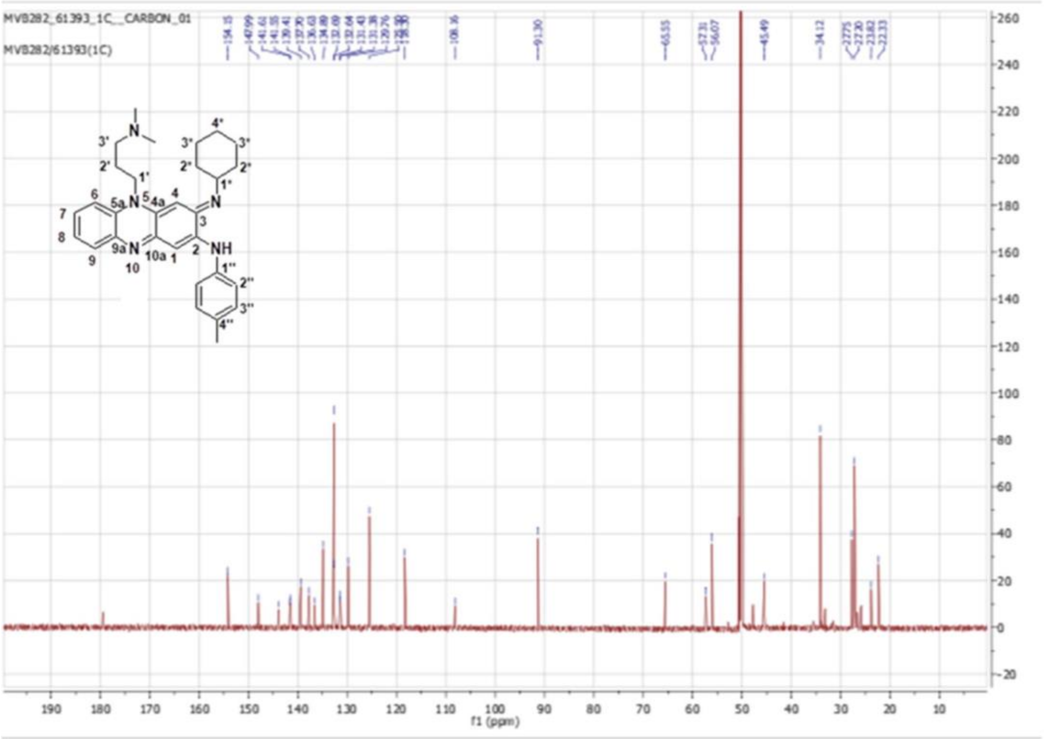

Figure S27: ^{13}C NMR of spectrum of 10p

A working stock solution of 10 μM of the test compound in fresh DMSO was prepared and stored at -70 °C until used. All compounds were tested at 5, 3, and 1 μM point concentrations for the initial screens. Mycobacterium tuberculosis H37Rv inocula were prepared from cultures grown on Lowenstein Jensen (LJ) slants. Mycobacterial suspensions were prepared in saline and the turbidity adjusted to 0.5 McFarland units. Aliquots of 100μl were inoculated into MGIT tubes and incubated at 37 °C until the inoculum became positive (about three days). 0.5 ml of positive MGIT cultures were transferred into MGIT tubes containing the test compounds. Controls consisting of a 1:100 dilution of the positive MGIT culture control in saline, 1.2% DMSO control in saline (concentration equivalent to that used when introducing the drug substance), and a 0.05 μg/ml isoniazid (INH) positive control in saline were also prepared. For mycobacterial growth evaluation, the MGIT 960 system (Becton Dickinson, Sparks, MD) was used, where M. tuberculosis growth is observed through fluorescent changes due to oxygen consumption during mycobacterial growth. Incubation at 37 °C was continued in the MGIT system, and the growth units (GU) were recorded hourly. For MIC99 evaluations, the 1% bacterial control culture was used; the MIC99 of the compound was determined relative to the growth units of the control (GU_400). When the GU of the control reached 400, the results were interpreted. If the drug-containing tube showed GU > 400, the material was defined as inducing resistance; if it showed GU ≤ 400, susceptibility of the mycobacteria to the drug substance was indicated at that concentration of drug substance. Compounds that showed GU = 0 at 1μM were considered to have an MIC ≤ 1 μM, and further MIC determinations on these materials using concentrations ranging from 0.0625 - 1 μM were performed. The analyses were done in triplicate and the averages are reported.

Table 1: MIC raw data and their statistical values.

**Sample Number**		**Concentration (μM)**		
		**1**	**3**	**5**
		Growth Units (GU)		
**10l**	< 1	0	837	478
**10m**	<1	0	37	381
**10h**	3	3640	2	35
**10g**	5	2774	2123	1345
**10c**	5	2120	3215	32
**10a**	3	3518	9	4
**10d**	3	2430	3777	2574
**10p**	<1	0	765	2438
**10o**	3	257	13	41
**10i**	5	3618	3148	172
**10b**	3	376	11	5
**10n**	3	260	26	3
**10j**	3	367	29	4
**10k**	5	261	122	743
**10e**	5	344	118	20
**10f**	3	3145	33	453
**Mean**		**481**	**297**	**182**

**Standard deviation**		**501**	**452**	**283**
**% range of SD from mean**		**104**	**152**	**155**

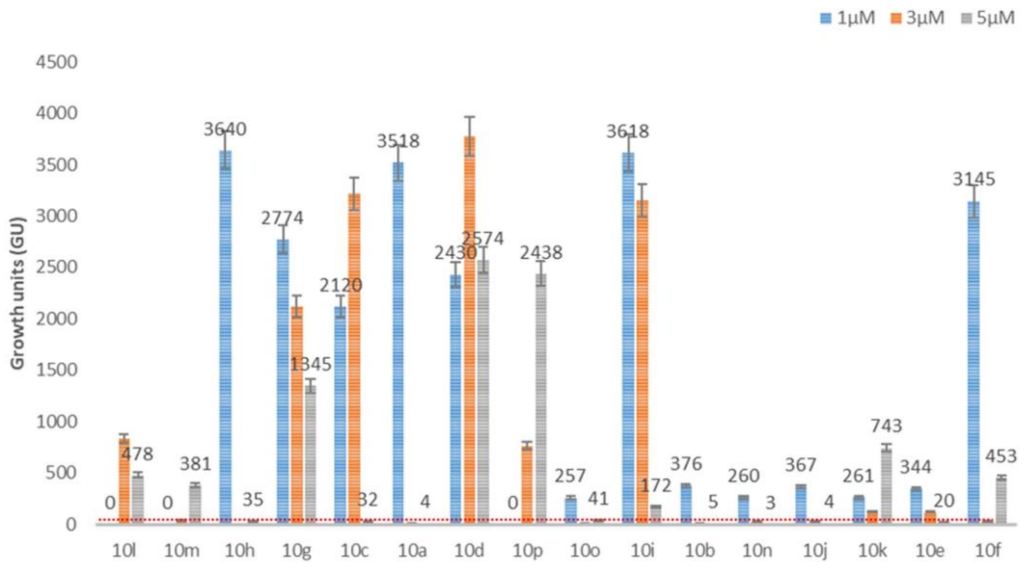

Figure S28: Primary activities of riminophenazine derivatives against H37Rv strain. Compounds 10l, 10m and 10p showed no growth units at 1 µM (red dotted line). Data are means of 3 biological repeats (n=3) performed in technical triplicates, error bars indicate SEM.

IN VITRO CYTOTOXICITY ASSAY

The WI-38 cell line - normal Human Fetal Lung Fibroblast from ECACC was routinely maintained as a monolayer cell culture at 37 °C, 5% CO_2, 95% air and 100% relative humidity in EMEM containing 10% fetal bovine serum, 2 mM L-glutamine and 50µg/ml gentamicin. For screening experiment, the cells (21-50 passages) were inoculated in a 96-well microtiter plates at plating densities of 10 000 cells/well and were incubated for 24 h. After 24 h the cells were treated with the experimental drugs which were previously dissolved in DMSO and diluted in medium to produce 5 concentrations. The process was performed in triplicate and the data presented are means of 3 repeats performed in triplicates. Cells without drug addition served as control. The blank contains complete medium without cells. Parthenolide was used as a standard. The plates were incubated for 48 h after addition of the compounds. Viable cells were fixed to the bottom of each well with cold 50% trichloroacetic acid, washed, dried and dyed by SRB. Unbound dye was removed and protein-bound dye was extracted with 10mM Tris base for optical density determination at the wavelength 540 nm using a multiwell spectrophotometer. Data analysis was performed using GraphPad Prism software. 50% of cell growth inhibition (IC50) was determined by non-linear regression.

Z’ factor: 0.9
IC50 : 18.5 µM

Concentration (µM)	Log Conc.	%Viability	SD
100	2.000	16.94	0.68
10	1.000	66.48	1.79
1	0.000	97.14	1.82
0.1	-1.000	101.92	0.99
0.01	-2.000	100.97	0.19

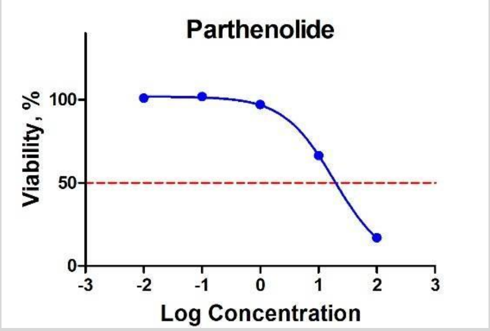

Figure S29: Data representing the control Parthenolide

10n
Z’ factor: 0.9
IC50 : 3.8 µM

Concentration (µM)	Log Conc.	%Viability	SD
100	2.000	22.12	4.57
10	1.000	37.42	2.40
1	0.000	87.02	0.69
0.1	-1.000	100.90	1.75
0.01	-2.000	97.20	3.08

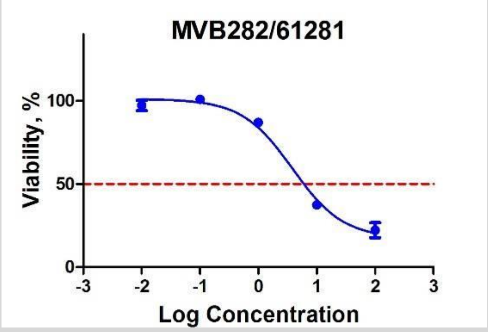

Figure S30: Data representing IC50 of compound 10n.

Reference

1. Micco, I.; Nencini, A.; Quinn, J. Bothmann H.; Ghiron, C.; Padova, A. *et al*. Parallel synthesis of a series of potentially brain penetrant aminoalkyl benzoimidazoles. *Bioorganic Med Chem*. **2008**, *16*: 2313–28.

2. Zhang, D.; Liu,Y.; Zhang, C.; Zhang, H.; Wang B.; Xu J. *et al*. Synthesis and biological evaluation of novel 2-methoxypyridylamino- substituted riminophenazine derivatives as antituberculosis agents. *Molecules*. **2014**, 19: 4380–94.
